# Brain-region-specific lipid dysregulation in L-DOPA-induced dyskinesia in a primate model of Parkinson’s disease

**DOI:** 10.1038/s41531-025-01109-6

**Published:** 2025-08-23

**Authors:** Ibrahim Kaya, Theodosia Vallianatou, Anna Nilsson, Patrik Bjärterot, Reza Shariatgorji, Per Svenningsson, Erwan Bezard, Per E. Andrén

**Affiliations:** 1 Department of Pharmaceutical Biosciences, Spatial Mass Spectrometry, Science for Life Laboratory, Uppsala University, Uppsala, Sweden; 2 Section of Neurology, Department of Clinical Neuroscience, Karolinska Institutet, Stockholm, Sweden; 3 Université de Bordeaux, Institut des Maladies Neurodégénératives, Bordeaux, France; 4 CNRS, Institut des Maladies Neurodégénératives, Bordeaux, France; 5 Motac Neuroscience, Bordeaux, France

**Keywords:** Parkinson's disease, Neurodegeneration

## Abstract

L-DOPA-induced dyskinesia (LID) is a significant and treatment-limiting complication in Parkinson’s disease (PD) therapy, yet its mechanisms remain poorly understood. We used high-resolution mass spectrometry imaging to map brain-region-specific alterations of glycerophospholipids and sphingolipids in a female macaque model of PD with and without LID following chronic L-DOPA treatment. LID was associated with depletion of antioxidant plasmalogen phosphatidylcholines in the globus pallidus interna, claustrum, and precentral gyrus—regions critical for motor function—and elevations of polyunsaturated fatty acid-containing glycerophospholipids, indicative of increased membrane fluidity. This lipid profile differed from similarly treated non-dyskinetic animals, suggesting lipid composition mediates differential susceptibility to LID. Lipid alterations correlated strongly with dyskinesia severity, dopamine, and L-DOPA concentrations, supporting a mechanistic link between lipid metabolism, neurotransmitter dysregulation, and LID. This comprehensive spatial lipidomic analysis identifies region-specific lipid dysregulation as a novel aspect of LID pathology, highlighting lipid pathways as potential therapeutic targets for mitigating dyskinesia.

## Introduction

Parkinson’s disease (PD) is a progressive neurodegenerative disease and the most common cause of a movement disorder characterized by motor symptoms resulting from degeneration of the dopaminergic nigrostriatal pathway, which disrupts normal functioning of the basal ganglia^1^. PD motor symptoms are effectively reversed by L-DOPA treatment, the precursor to dopamine^2^. However, although L-DOPA treatment is highly effective in the early stages of PD, its prolonged use can cause motor complications, including involuntary movements, termed L-DOPA-induced dyskinesia (LID). LID can become treatment-limiting^2,3^. Therefore, to prevent dyskinesia, it is important to understand the molecular mechanisms involved in LID.

Lipids are being increasingly considered as a prominent class of metabolites in neuroscience and neurodegenerative disease research^4–6^. The main roles of lipids in cell function were initially thought to be as structural components of membranes and energy repositories. However, recent studies have indicated that lipids have critical functions in mediating intracellular signalling, inflammation, cell growth and polarity, homoeostasis, maintenance and cell senescence^4,6,7^. Therefore, lipidomics has become an important metabolomics research pathway for studying neurodegeneration.

Evidence has been acquired that strongly links lipids to many aspects of PD^8,9^, including α-synuclein pathology^8,10,11^, and genetic risk factors, which feature mutations in genes involved in lipid metabolism, such as *GBA1*, *VPS35* and *PINK1*^12,13^. Although several lipidomics studies have shown altered lipid levels in post-mortem PD patient brains^14–17^ and rodent and primate PD model brains^18,19^, there are only a few reports on LID-specific changes. Previous metabolomics studies have reported LID-specific alterations of glycerophospholipids and endocannabinoid signalling in dyskinetic rodent models^20^ and significant dysregulation in glycosphingolipid metabolism in plasma and cerebrospinal fluid (CSF) samples from LID patients, correlating closely with the severity of dyskinetic movements^21^. While these clinical findings underscore the relevance of lipid pathway dysregulation in LID, they do not report detailed changes at the individual lipid species level. Nevertheless, the consistent implication of altered lipid pathways in both clinical and preclinical LID studies strengthens the translational potential of targeting lipid metabolism therapeutically.

Furthermore, several reports have indicated the therapeutic use of certain lipid or lipid precursor molecules that reduce LID in animal LID models, including plasmalogen precursors^22–24^, docosahexaenoic acid (DHA)^25–27^ and endocannabinoids^28^. However, these studies did not provide a global analysis of lipid alterations within different spatial locations in brain samples. Therefore, thorough in situ regional and subregional analyses of brain lipid molecules are needed to clarify the role of lipids in LID and facilitate further research on the therapeutic potential of lipids for the treatment of LID and PD.

While most lipidomic approaches lack anatomical specificity, mass spectrometry imaging (MSI) is a powerful technique for examining distributions of a wide range of lipid molecules within tissue sections^29^. Its utility in neurodegeneration and neuropharmacodynamic research is well-established^30,31^. Due to its versatility and rapid improvements in analytical capabilities, matrix-assisted laser desorption/ionization (MALDI)-based MSI has been widely used to investigate region-specific lipid changes associated with neurodegenerative diseases^32^.

Animal models have provided fundamental insights into brain aging and neurodegeneration-related brain function alterations^33,34^. Using MALDI-MSI, we previously imaged and investigated regional alterations in neurotransmitters, their associated metabolites^35,36^ and neuropeptides^37^ in coronal brain tissue sections of a 1-methyl,4-phenyl-1,2,3,6 tetrahydropyridine (MPTP) administered non-human primate model of PD^38^ during peak-dose LID^39^. MPTP-treated PD models closely mimic the clinical symptoms and several aspects of disease pathology of PD and LID^40,41^. Furthermore, we recently reported a MALDI-MSI study of hydroxylated and non-hydroxylated sulfatide lipid distributions within non-human primate brains and their specific alterations within specific brain regions in MPTP-lesioned non-human primate brains^42^. We also used MALDI-MSI to examine brain-region-specific alterations of glycerophospholipids and sphingolipids in dopaminergic and serotonergic prosaposin (PSAP)-deficient mouse models, revealing information on the role of PSAP in the maintenance of lipid metabolism in dopaminergic neurons^43^. Therefore, spatial lipidomics via MSI is a powerful tool for dissecting the region-specific roles of lipids in PD brains.

In the present study, we analysed samples from the same pre-existing cohort of female non-human primate brains (*Macaca mulatta*) representing four groups: (a) control animals (Ctrl); (b) animals with parkinsonism induced by MPTP administration (MPTP); (c) MPTP-treated animals that received chronic L-DOPA treatment without dyskinesia (non-LID), and (d) animals exhibiting LID (LID)^39,44,45^. We utilized high mass resolution MALDI-Fourier-transform ion cyclotron resonance (FTICR)-MSI in dual polarity (negative and positive ionization) modes^46–48^ to map the distributions of several glycerophospholipids and sphingolipids in coronal brain tissue sections. The results revealed several brain-region-specific alterations of these lipids that were specific to both MPTP and LID within coronal brain tissue sections. In addition, we investigated correlations between the levels of altered lipids and dopamine transporter (DAT) binding of MPTP-lesioned animals, LID behavioural scores of L-DOPA-treated animals and dopamine and L-DOPA levels within specific brain regions. Overall, this study provides significant insights into the role of lipids in PD and LID, and sheds further light on the therapeutic potential of lipid treatments.

## Results

### Region-specific untargeted data analysis

**Fig. 1:**
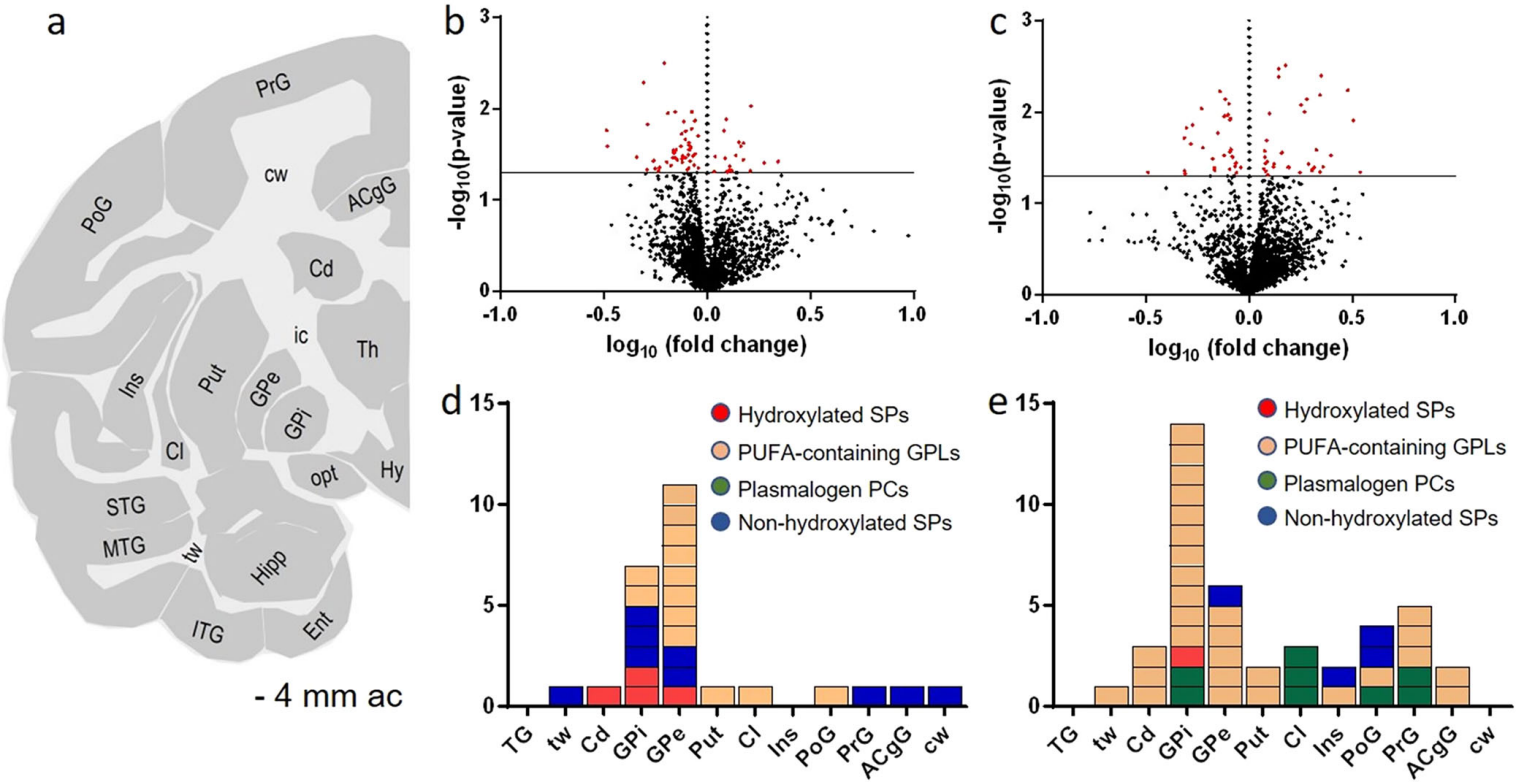
Region-specific untargeted lipidomics analysis of MALDI-FTICR-MSI data in a non-human primate model of Parkinson’s disease and L-DOPA-induced dyskinesia. Untargeted region-specific lipidomics workflow designed to identify lipid changes displaying differences between Ctrl and MPTP in GPi and GPe brain regions and between non-LID and LID in GPi, GPe, Put, Cd, PrG, PoG, TG, Cl, tw, cw, Ins and ACgG brain regions. **a** Schematic of a coronal non-human primate brain tissue section at −4 mm from the ac depicting different white and grey matter brain regions. Untargeted mass lists including 4879 *m/z* values obtained in dual polarity mode from each analysed brain region were extracted and evaluated by hypothesis testing using multiple two-tailed *t*-tests. Representative volcano plots showing significantly altered *m/z* values in red between **b** Ctrl and MPTP and **c** non-LID and LID groups in the GPi brain region. Identified lipid molecules showing significant changes were grouped into four different classes: (i) plasmalogen phosphatidylcholines (PCs), (ii) polyunsaturated fatty acid (PUFA)-containing glycerophospholipids (GPLs), (iii) hydroxylated sphingolipids (SPs), and (iv) non-hydroxylated SPs. Lower panels: panels **d** and **e** show coloured stacked bars indicating how many lipid species from each class exhibited significant changes between **d** Ctrl and MPTP, and **e** non-LID and LID in different brain regions. PoG postcentral gyrus, PrG precentral gyrus, STG superior temporal gyrus, ACgG anterior cingulate gyrus, MTG middle temporal gyrus, ITG inferior temporal gyrus, Ent entorhinal area, Hipp hippocampus, Cd caudate nucleus, Ins insula, opt optical tract, Cl claustrum, Put putamen, GPe/GPi globus pallidus externa/interna, Hy hypothalamus, Th thalamus, ic internal capsule, tw temporal white matter, cw cerebral white matter.

Lipidomic analysis was performed using MALDI-MSI in both negative and positive ionization modes on coronal brain sections of Ctrl, MPTP, non-LID and LID groups. Sections were taken at −4 mm relative to the anterior commissure (ac)^49^ (Fig. 1a), allowing investigation of motor-related brain areas. e.g., the caudate (Cd), putamen (Put), precentral gyrus (PrG, where the primary motor cortex is located) and internal and external segments of the globus pallidus (GPi and GPe, respectively). Other discrete cortical and white matter brain regions were also analysed, including the postcentral gyrus (PoG), temporal gyrus (TG) - which combines the superior (STG), middle (MTG), and inferior (ITG) temporal gyri -, along with the insula (Ins), claustrum (Cl), anterior cingulate gyrus (ACgG), temporal white matter (tw) and cerebral white matter (cw) (Fig. 1a).

From the dual polarity MALDI-MSI data, we generated an untargeted mass list including 4879 *m/z* values for each analysed brain region, and corresponding peak area values were used to perform multiple two-tailed *t*-tests comparing Ctrl vs. MPTP and non-LID vs. LID; results are shown in the volcano plots in Fig. 1b and c, respectively.

We previously investigated spatial lipid alterations within coronal brain sections taken at −6 mm relative to the ac in the same individual animals, focusing on basal ganglia regions, including the GPi, GPe and substantia nigra reticulata (SNR), and reported specific distributions and alterations in hydroxylated and non-hydroxylated sulfatides in control and MPTP primates^42^. The current study focused on delineating lipid alterations specific to LID. We recently demonstrated that LID was linked to dysregulation of L-DOPA metabolism throughout the brain, showing that L-DOPA was highly elevated in LID animals, leading to increased DA and DA metabolites in extrastriatal areas. In addition, 3-*O*-methyldopa (3-OMD), the main metabolite of L-DOPA, was highly elevated in LID^35^.

In the present study, we annotated and investigated the twelve brain regions described above for non-LID vs. LID group analysis. Multiple *m*/*z* values contributing to group separation were selected for identification (Supplementary Table 1). Accurate mass matching to databases and literature, followed by on-tissue tandem MS (MS/MS) analysis, revealed alterations in four main lipid categories (Supplementary Table 2): (i) plasmalogen PC, (ii) polyunsaturated fatty acid (PUFA)-containing GPLs, (iii) hydroxylated sphingolipids, and (iv) non-hydroxylated sphingolipids (Fig. 1d, e).

### Changes in plasmalogen phosphatidylcholines

**Fig. 2:**
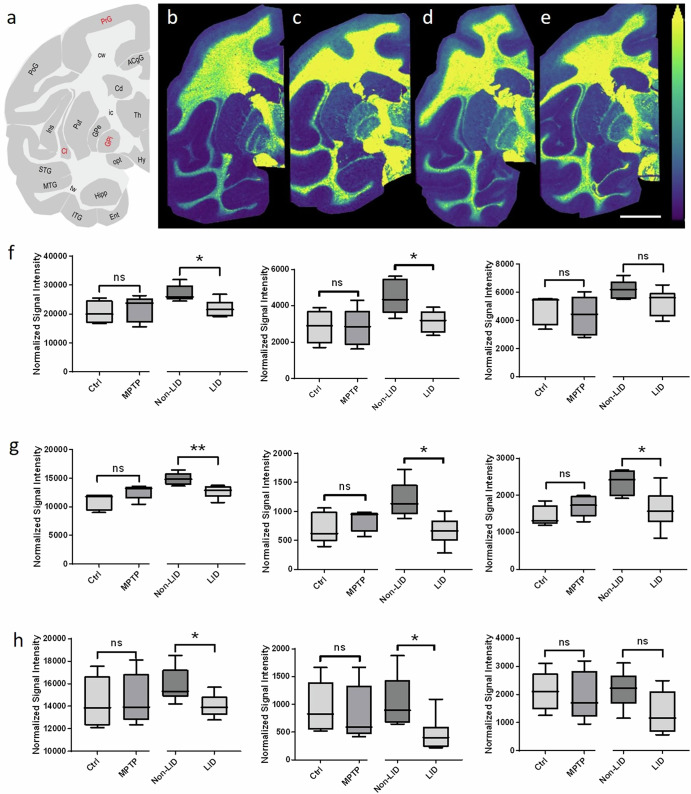
Brain-region-specific changes in plasmalogen PCs between non-LID and LID groups. **a** Schematic of a coronal non-human primate brain tissue section at -4 mm from the ac depicting different brain regions, with those showing changes in plasmalogen PCs between non-LID and LID groups labelled in red. Representative ion images of the [M + H]^+^ ion of PC-P (34:0) in **b** Ctrl, **c** MPTP, **d** non-LID, and **e** LID brain tissue sections at −4 mm from the ac. All ion images are scaled to the maximum intensity of the individual ion. The ion image is RMS-normalized in panels **b**–**e**. Lateral resolution: 150 µm; scale bar: 9 mm. Results of statistical analysis using Student’s *t*-test of plasmalogen PCs (from left to right) PC-P(34:0), PC-P(36:0) and PC-P(36:1) in **f** GPi, **g** Cl, and **h** PrG brain regions were performed using. Changes were statistically evaluated between Ctrl and MPTP, and non-LID and LID, independently. Asterisks indicate significance: *P < 0.05; **P < 0.01; ns: not significant.

A notable finding was the differential regulation of plasmalogen PC species between LID and non-LID animals. In the GPi, Cl, PoG and PrG, significant changes in plasmalogen PC levels were observed (Fig. 1e). MALDI-MSI analysis (Fig. 2a–e) of Ctrl, MPTP, non-LID and LID animals revealed the ion distribution of PC-P (34:0) ([M + H]^+^), showing higher abundance within white matter brain regions (Fig. 2b–e). Comparative analyses of PC-P(34:0), PC-P(36:0) and PC-P(36:1) between the groups (Ctrl vs. MPTP and non-LID vs. LID) indicated decreased plasmalogen PC levels in LID animals compared to non-LID animals within the GPi, Cl and PrG brain regions (Fig. 2f–h). These trends were consistent across multiple brain regions, as shown in heat maps of *z*-scores for plasmalogen PC in the GPi, GPe, Put, Cl, Cd and PrG (Supplementary Fig. 1). However, there was no apparent decrease of plasmalogen PCs in LID compared to non-LID animals in the Cd (Supplementary Fig. 1b).

### Changes in PUFA-containing glycerophospholipids

**Fig. 3:**
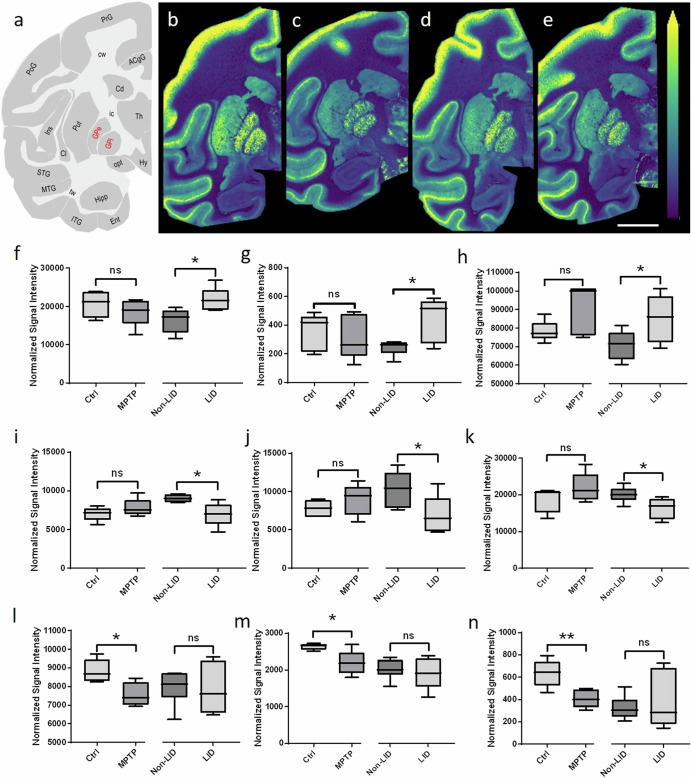
Brain-region-specific alterations in PUFA-containing GPLs between Ctrl and MPTP, and non-LID and LID groups. **a** Schematic of a coronal non-human primate brain tissue section at −4 mm from the ac depicting different brain regions, with those evaluated labelled in red. Representative images of the [M-H]^−^ ion of PI (40:6) in (from left to right) **b** Ctrl, **c** MPTP, **d** non-LID, and **e** LID brain tissue sections at −4 mm from the ac. All ion images are scaled to the maximum intensity of the individual ion. The ion images are RMS-normalized in panels **b**–**e**. Lateral resolution: 150 μm; scale bar: 9 mm. Results of statistical analysis using Student’s *t*-test of PUFA-containing GPLs **f** PI(40:6), **g** PI(40:7), **h** PC (36:4), **i** PC(38:3), **j** PS (38:3) and **k** PC(36:3) in the GPi, and **l** PE(40:6), **m** PC(40:7), and **n** PC(42:7) in the GPe. Changes were statistically evaluated between Ctrl and MPTP, and non-LID and LID, independently. Asterisks indicate significance: *P < 0.05; **P < 0.01; ns: not significant.

Levels of specific PUFA-containing GPLs differed between Ctrl and MPTP animals and between the non-LID and LID groups. Significant alterations were particularly evident in the GPi and GPe regions of the basal ganglia (Fig. 1d, e). MALDI-MSI analysis (Fig. 3a–e) of Ctrl, MPTP, non-LID and LID animals revealed that the ion distribution of PI(40:6) ([M-H]^−^) was predominantly localized within the grey matter brain regions (Fig. 3b–e). Statistical comparisons of GPL levels among these groups were performed. To illustrate the patterns of PUFA-containing GPL changes in the GPi and GPe, heat maps of *z*-scores were generated (Supplementary Fig. 2). Ion distributions of the additional GPLs were also visualized in control non-human primate brain tissue sections (Supplementary Fig. 3).

Three main trends in GPL alterations emerged across the groups in the GPi and/or GPe brain regions: (i) Some PUFA-containing GPLs, including PI(40:6), PI(40:7) and PC(36:4), were higher in LID animals compared to non-LID animals (Fig. 3f–h); (ii) Some GPLs, including PC(38:3), PS(38:3), and PC(36:3), were lower in LID animals compared to non-LID animals (Fig. 3i–k); and (iii) some PUFA-containing GPLs, including PE(40:6), PC(40:7) and PC(42:7), were lower in MPTP animals compared to Ctrl animals (Fig. 3l–n).

### Relationships between the changes of plasmalogen phosphatidylcholines, PUFA-containing glycerophospholipids and severity of dyskinesia

**Fig. 4:**
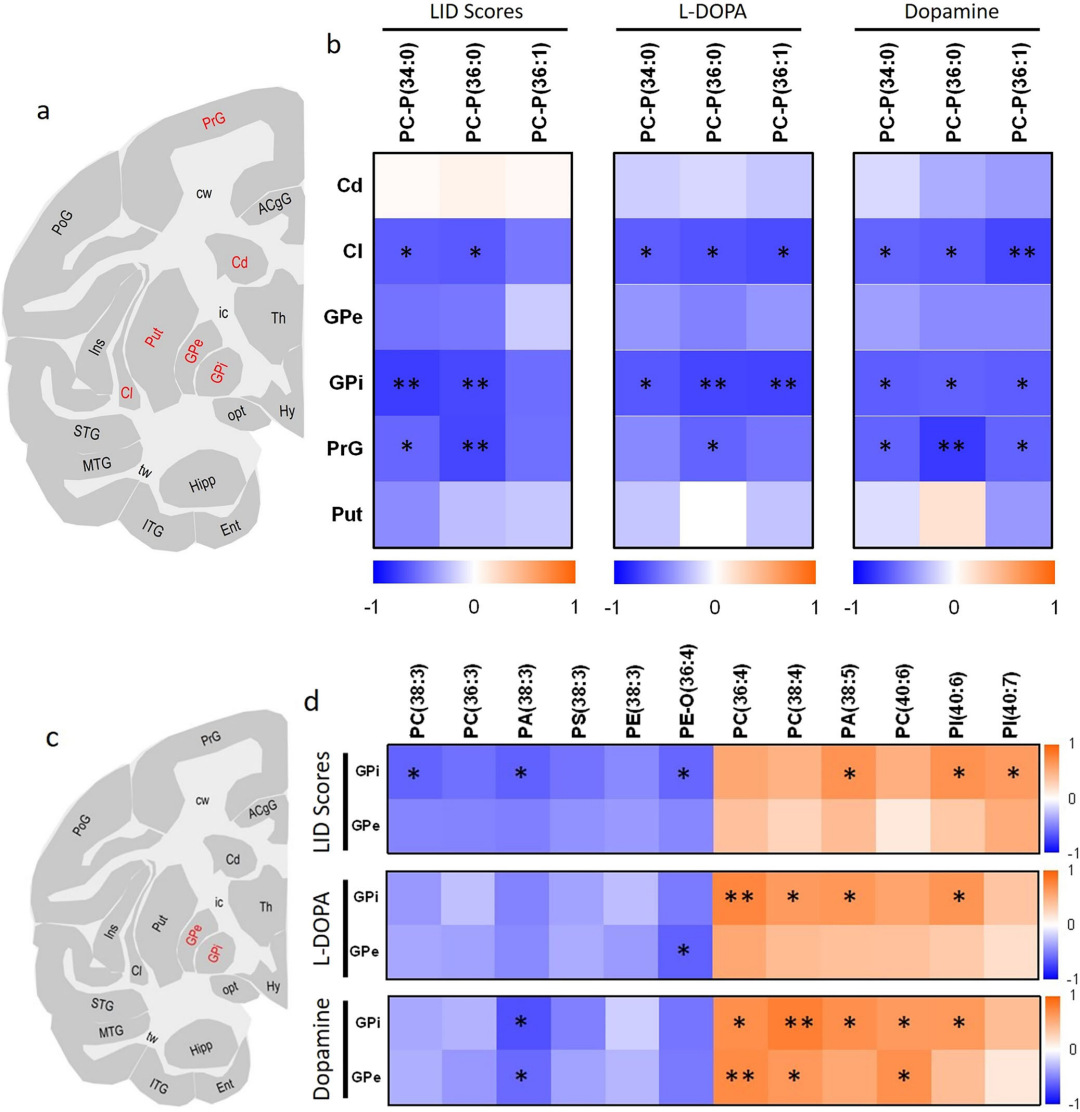
Correlation of LID scores, L-DOPA levels and dopamine levels with plasmalogen PCs and PUFA-containing glycerophospholipids in specific brain regions. **a** Schematic of a coronal non-human primate brain tissue section at −4 mm from the ac depicting different brain regions, with those evaluated labelled in red. **b** Results of Pearson’s correlation analysis between plasmalogen PC levels in the GPi, GPe, Put, Cd, Cl and PrG, and LID scores of the animals or levels of L-DOPA and dopamine obtained from the same regions of consecutive tissue sections. **c** Schematic of a coronal non-human primate brain tissue section at −4 mm from the ac depicting different regions, with those evaluated labelled in red. **d** Results of Pearson’s correlation analysis between glycerophospholipid levels in the GPi and GPe, and LID scores of the animals, as well as levels of L-DOPA and dopamine obtained from the same regions in consecutive tissue sections. Heat maps are colour-coded according to Pearson’s correlation coefficients: orange and blue colours indicate positive and negative correlations, respectively. Asterisks indicate significant correlations: *P < 0.05; **P < 0.01.

To further investigate relationships between plasmalogen PCs and the severity of dyskinesia, we examined correlations between the abundances of plasmalogen PCs in specific brain regions and dyskinesia behavioural scores of the animals, along with L-DOPA and dopamine levels obtained from the same regions of consecutive brain tissue sections (Fig. 4a, b). Significant correlations were observed between PC-P(34:0) and PC-P(36:0) levels and dyskinesia scores in the GPi, Cl and PrG. Further, significant correlations were observed between PC-P(34:0), PC-P(36:0) and PC-P(36:1) levels and L-DOPA levels within the GPi and Cl, with PC-P(36:0) also showing a strong correlation in the PrG. In line with this, PC-P(34:0), PC-P(36:0), and PC-P(36:1) levels correlated significantly with dopamine concentrations within the GPi, Cl and PrG (Fig. 4b). These findings indicate a negative correlation between plasmalogen PC levels and LID severity scores and L-DOPA and dopamine levels in the GPi, Cl and PrG, suggesting that plasmalogen PC depletion may be linked to the susceptibility to LID in these brain regions.

To further investigate the relationships between changes in GPL levels between LID and non-LID animals and dyskinesia severity, we examined correlations between the abundances of specific GPLs in motor-related regions and dyskinesia scores, as well as L-DOPA and dopamine levels from adjacent tissue sections (Fig. 4c, d). Briefly, PUFA-containing GPLs with a higher degree of saturation, which were decreased in LID animals compared to non-LID animals, showed a negative correlation with LID scores and L-DOPA and dopamine levels (Fig. 4d). In contrast, more unsaturated PUFA-containing GPLs, which were elevated in LID compared to non-LID animals, demonstrated positive correlations with LID scores and L-DOPA and dopamine levels (Fig. 4d). Further, PUFA-containing GPLs that showed a decreasing trend in MPTP relative to Ctrl animals positively correlated with DAT binding scores in the Cd and Put (Supplementary Fig. 4).

### Changes in sphingolipids

**Fig. 5:**
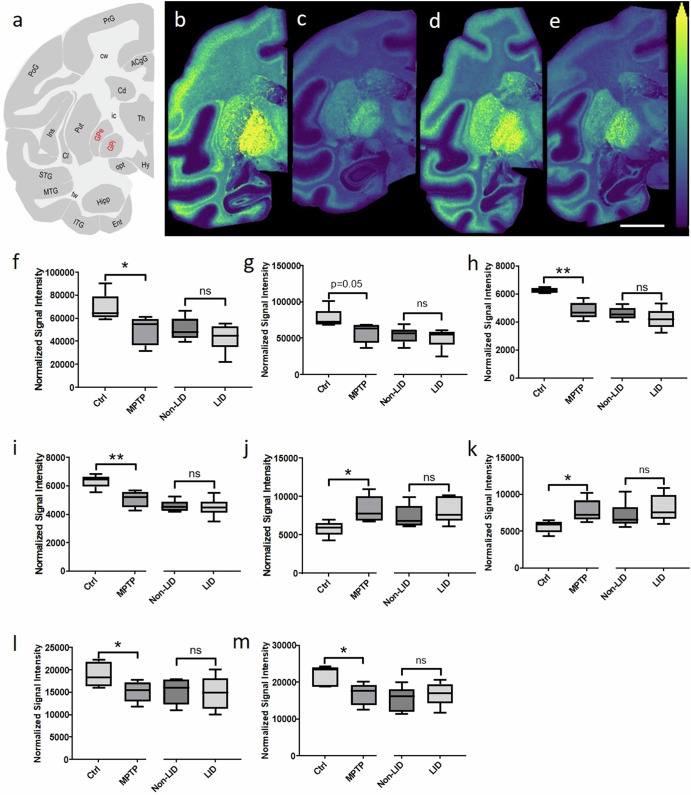
Brain-region-specific changes in sphingolipids between Ctrl and MPTP groups. **a** Schematic of a coronal non-human primate brain tissue section at −4 mm from the ac depicting different brain regions, with those evaluated labelled in red. Representative images of the [M-H]^−^ ion of SHexCer(t43:2) in (from left to right) **b** Ctrl, **c** MPTP, **d** non-LID, and **e** LID brain tissue sections at −4 mm from the ac. All ion images are scaled to the maximum intensity of the individual ion. The ion image is RMS-normalized in panels **b**–**e**. Lateral resolution: 150 µm; scale bar: 9 mm. Results of statistical analysis using Student’s *t* test of hydroxylated and non-hydroxylated sphingolipids SHexCer(t43:2) in **f** the GPi and **g** the GPe, HexCer(t42:2) in **h** the GPi and **i** the GPe, SHexCer(d40:1) in **j** the GPi and **k** the GPe, and SM (d43:2) in **l** the GPi and **m** the GPe. Changes were statistically evaluated between Ctrl and MPTP, and non-LID and LID, independently. Asterisks indicate significance: *P < 0.05; **P < 0.01; ns: not significant.

Levels of some long-chain hydroxylated and non-hydroxylated sphingolipid species differed between the MPTP and Ctrl groups, with notable differences observed in the GPi and GPe regions of the basal ganglia (Fig. 1d). MALDI-MSI analysis (Fig. 5a–e) of the Ctrl, MPTP, non-LID and LID groups revealed the distribution of SHexCer(t43:2) ([M-H]^−^), which was more abundant in white matter-rich areas and in basal ganglia regions, including the GPi, GPe, Put and Cd (Fig. 5b–e). Changes in levels of SHexCer(t43:2), HexCer(t42:2), SHexCer(d40:1) and SM(d43:2) were analysed between groups, and significant alterations were observed. MPTP animals showed a decrease in hydroxylated hexosylceramides, including SHexCer(t43:2) (Fig. 5f, g) and HexCer(t42:2) (Fig. 5h, i), along with a increase in non-hydroxylated SHexCer(d40:1) (Fig. 5j, k) and an decrease in non-hydroxylated SM(d43:2) (Fig. 5l, m) in the GPi and GPe.

**Fig. 6:**
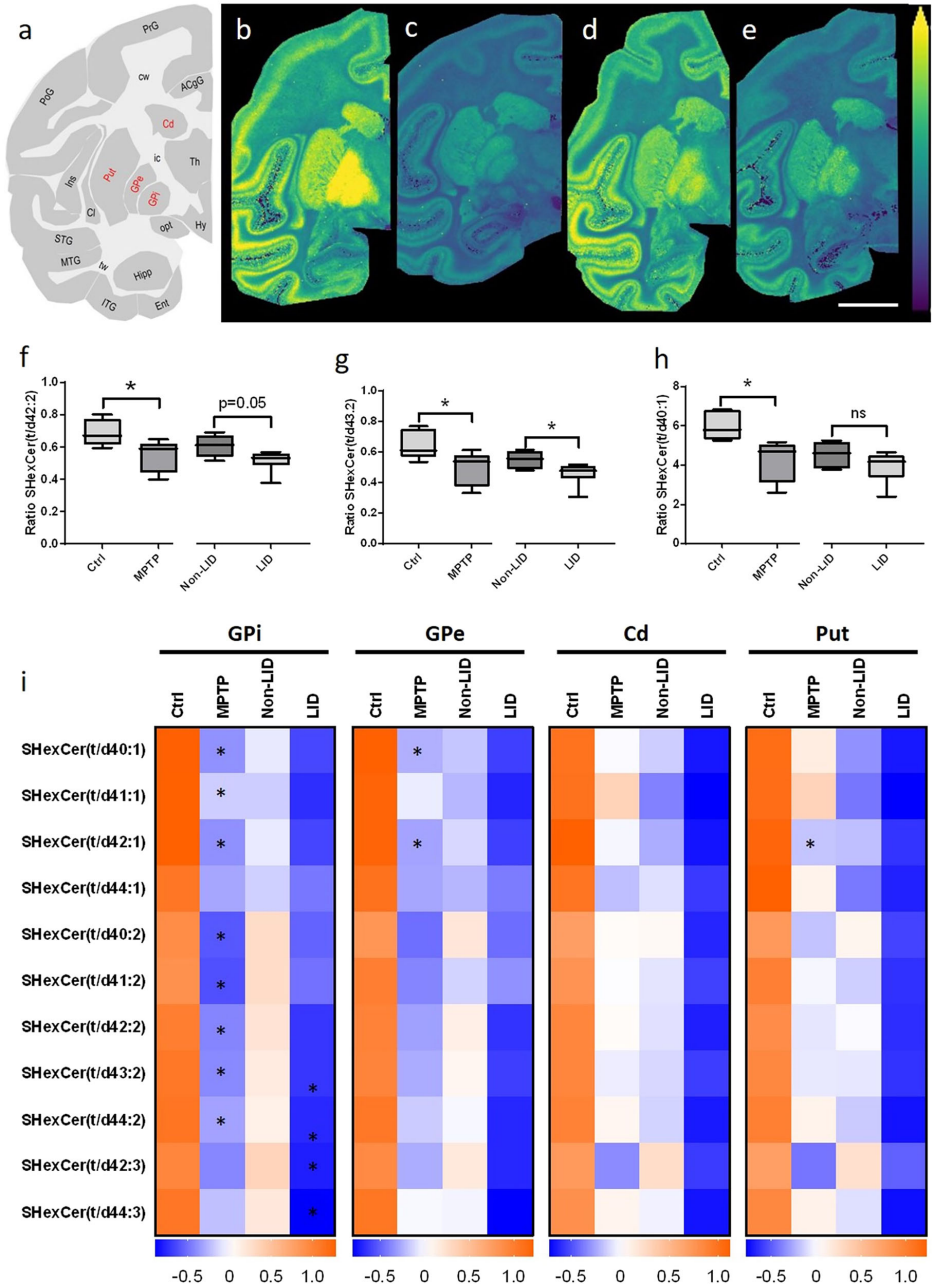
Brain-region-specific changes in the ratios of hydroxylated to non-hydroxylated (t/d) SHexCers between Ctrl and MPTP, and non-LID and LID groups. **a** Schematic of a coronal non-human primate brain tissue section at −4 mm from the ac depicting different brain regions, with those evaluated labelled in red. Representative images of the [M-H]^−^ ion of SHexCer(t42:2) normalized to the [M-H]^−^ ion of SHexCer(d42:2) in (from left to right) **b** Ctrl, **c** MPTP, **d** non-LID, and **e** LID brain tissue sections at −4 mm from the ac. All ion distribution images are scaled to the maximum intensity of the individual ion. The ion images are RMS-normalized in panels **b**–**e**. Lateral resolution: 150 µm; scale bar: 9 mm. Results of statistical analysis using Student’s *t*-test of **f** SHexCer(t/d42:2), **g** SHexCer(t/d43.2), and **h** SHexCer(t/d40:1) in the GPi. Changes were evaluated between Ctrl and MPTP, and non-LID and LID. **i** Heat maps showing *z*-scores of several detected long-chain SHexCer(t/d) in the GPi, GPe, Cd and Put basal ganglia brain regions. Changes were statistically evaluated between Ctrl and MPTP, and non-LID and LID, independently. Asterisks indicate significance: *P < 0.05; **P < 0.01; ns: not significant. Asterisks in the MPTP and LID columns show results of statistical analysis between Ctrl vs. MPTP groups and LID vs. non-LID groups, respectively.

To further explore changes between long-chain hydroxylated and non-hydroxylated SHexCers, we examined heat maps of *z*-scores in the GPi and GPe brain regions (Supplementary Fig. 5), which revealed a general trend of decreased hydroxylated SHexCers and increased non-hydroxylated SHexCers in MPTP compared to Ctrl animals. Interestingly, a similar trend was seen between LID and non-LID animals, with LID showing reduced hydroxylated SHexCers and elevated non-hydroxylated SHexCers (Supplementary Fig. 5b). We also evaluated ratios of hydroxylated to non-hydroxylated SHexCers across Ctrl, MPTP, non-LID and LID animals in different brain regions (Fig. 6a–e). MALDI-MSI analysis (Fig. 6a–e) of the Ctrl, MPTP, non-LID and LID groups revealed the distribution of normalized hydroxylated to non-hydroxylated SHexCer(t/d42:2) ([M-H]^−^) in basal ganglia regions, including the GPi, GPe, Put and Cd (Fig. 6b–e). We found that MPTP animals had significantly lower ratios of SHexCer(t/d42:2), SHexCer(t/d43:2) and SHexCer(t/d40:1) in GPi compared to controls, with similar depletion observed in LID animals compared to non-LID animals (Fig. 6f–h). Heat maps of these ratios in the GPi, GPe, Put, and Cd (Fig. 6i) confirmed a consistent pattern of reduced t/d ratios of SHexCers in MPTP and LID animals (Fig. 6i). Interestingly, PUFA-containing SHexCers showed elevated ratios in non-LID compared to MPTP animals, whereas mono-unsaturated SHexCers did not exhibit the same pattern in the GPi and GPe (Fig. 6i).

To investigate the relationship between specific sphingolipid changes and PD or LID severity, we examined correlations between sphingolipid levels and DAT binding in the Cd and Put of Ctrl and MPTP animals, as well as correlations between sphingolipid levels and dyskinesia scores and L-DOPA and dopamine levels in basal ganglia regions. In MPTP animals, depletion of HexCer(t42:2), SHexCer(t43:2), and SM(d43:2) and an increase in SHexCer(d40:1) in the GPi and GPe correlated significantly with DAT scores in the Cd and Put (Supplementary Figs. 6 and 7). Targeted analysis of hydroxylated and non-hydroxylated sulfatides revealed that depletion of hydroxylated SHexCers in the GPi showed a trend of negative correlation with L-DOPA levels (Supplementary Fig. 7). Interestingly, several hydroxylated and non-hydroxylated sulfatides displayed strong negative correlations with dopamine levels in the Put (Supplementary Fig. 7). Further correlations were noted in the GPi and GPe, i.e., hydroxylated sulfatides were positively correlated and non-hydroxylated sulfatides were negatively correlated with DAT binding in the Cd and Put (Supplementary Fig. 7).

Lastly, ratios of hydroxylated to non-hydroxylated sulfatides showed strong negative correlations for PUFA-containing sulfatides with L-DOPA levels in the GPi and dopamine levels in the Put (Supplementary Fig. 8). Further, the hydroxylated to non-hydroxylated ratios of several sulfatides in the GPi, GPe, and Put correlated positively with DAT binding in the Cd and Put, indicating potential links between lipid metabolism and neurotransmitter dynamics in PD and LID pathology (Supplementary Fig. 8).

## Discussion

This study provides important insights into the brain-region-specific lipidomic alterations associated with L-DOPA-induced dyskinesia in the gold standard MPTP female *Macaca mulatta* model of PD. Using MALDI-MSI, we identified significant dysregulation in multiple lipid classes, including plasmalogen PCs, PUFA-containing GPLs, hydroxylated sphingolipids and non-hydroxylated sphingolipids, within key motor-related brain regions. These findings contribute to our understanding of PD and LID’s molecular mechanisms, suggesting potential new therapeutic targets related to lipid metabolism and oxidative stress.

Firstly, we found that plasmalogen PCs were depleted in LID compared to non-LID animals in the GPi, Cl and PrG, whereas they were increased in non-LID compared to MPTP animals. This LID-specific depletion of plasmalogen PCs showed a strong correlation with LID scores as well as L-DOPA and dopamine levels in these regions.

Ether lipids, which include plasmalogens, constitute approximately 8–20% of the total glycerophospholipid content in humans. Plasmalogens are particularly abundant in the brain, where they play essential roles as structural components of subcellular membranes and are enriched in lipid rafts^50,51^. The synthesis of plasmalogens is initiated in peroxisomes and is completed in the endoplasmic reticulum^52^. Peroxisomal dysfunction is linked to several neurodegenerative diseases, including PD^53^. Plasmalogens are known for their anti-oxidant properties, acting as scavengers of reactive oxygen species (ROS)^54^ and reducing plasmalogen levels, thus increasing cellular susceptibility to ROS and promoting cell death^55^. Indeed, decreased plasmalogen levels have been shown to be associated with neurodegenerative diseases^56^, and they are being investigated as a treatment for conditions like PD^23,57^ and LID^22,24^.

Our data indicated that depletion of saturated and monounsaturated plasmalogen PCs in LID vs. non-LID animals within the GPi, Cl and PrG might indicate localized peroxisomal dysfunction induced by LID pathology. The basal ganglia’s direct pathway, which projects from the striatum to the GPi and then onward to the thalamus and motor cortices^3^, is thought to play a significant role in dyskinesia. Therefore, the observed plasmalogen deficiency in the GPi and associated PrG region, where the primary motor cortex resides, supports a link between dyskinesia-induced plasmalogen depletion and dysfunction in these motor circuits. Indeed, we observed a trend of plasmalogen depletion in the GPe and Put, indicating that plasmalogen deficiency may contribute to broader basal ganglia dysfunction in LID.

The Cl, a thin sheet of grey matter subcortical structure comprised of neurons in the forebrain, is thought to have extensive connectivity with the cerebral cortex and may serve as a limbic-sensory-motor interface^58^. Some evidence links the pathology in the claustrum with clinical symptoms seen in PD patients^59^. Here, we observed LID-specific depletion of plasmalogen PCs in the Cl, suggesting a potential role for this structure in the MPTP-induced PD pathology. Interestingly, plasmalogen precursors have been reported to alleviate LID in MPTP-induced PD models^22,24^, which agrees with our findings and highlights the therapeutic potential of targeting plasmalogens in LID.

Further, we found significant negative correlations between plasmalogen PC levels and LID scores and L-DOPA and dopamine levels in the GPi, PrG and Cl brain regions, suggesting that regional plasmalogen depletion may be associated with both the molecular pathology of LID and clinical severity of LID. The elevation of plasmalogen PCs in non-LID compared to MPTP animals within the basal ganglia regions (GPi, GPe, Cd, Put), as well as in the Cl and PrG, suggests an interaction between L-DOPA treatment and plasmalogen PC biochemistry. Specifically, elevated plasmalogen levels in non-LID animals may provide neuroprotective effects against oxidative stress induced by MPTP treatment. Conversely, subsequent depletion in LID animals could reflect increased oxidative stress susceptibility associated with dyskinesia pathology.

Secondly, we observed three distinct trends in PUFA-containing GPL changes within the GPi and GPe among the animal groups. Specific PUFA-containing GPLs were higher in LID animals than in non-LID animals and lower in non-LID animals compared to MPTP animals. More highly saturated PUFA-containing GPLs showed a decrease in LID animals compared to non-LID animals, and were increased in non-LID animals compared to MPTP animals. Some PUFA-containing GPLs were lower in MPTP animals than in Ctrl animals, specifically within the GPi and GPe brain regions. The LID-specific elevation of certain PUFA-containing GPLs positively correlated with LID scores, as well as with L-DOPA and dopamine levels. In contrast, less unsaturated PUFA-containing GPLs negatively correlated with these measures. Further, the MPTP-induced depletion of certain PUFA-containing GPLs positively correlated with DAT measurements in the Cd and Put.

GPLs have diverse molecular structures and are major constituents of neural membranes, with their turnover rates dependent on their distinct structure and localization differences across cells and membranes. Alterations in GPL composition can affect membrane fluidity, flexibility, thickness and permeability, influencing neural functions significantly^60,61^. GPLs also serve as reservoirs for second messengers and are involved in apoptosis. Modulation of transporters and membrane-bound enzyme activities^60^ and alterations of GPL composition of neural membranes have been reported for several neurodegenerative diseases^60^. Our findings indicate a depletion of PUFA-containing GPLs in MPTP compared to Ctrl animals within the GPi and GPe, with some GPL depletions, particularly in the GPe, correlating with DAT binding scores in the Put and Cd. Membranes rich in PUFA-containing GPLs are highly vulnerable to oxidative damage, especially as the degree of unsaturation increases^62^. Therefore, the depletion of PUFA-containing GPLs might result from oxidative damage by MPTP-induced dopaminergic neurodegeneration pathology.

Furthermore, we found that certain PUFA-containing GPLs, including DHA-containing species, increased in LID animals relative to non-LID animals, whereas more unsaturated species decreased. Converse trends were observed in non-LID compared to MPTP animals. This shift in GPL composition suggests that L-DOPA treatment and the onset of LID significantly alter the composition of cellular membrane structure. Increasing PUFA-containing GPLs and reducing saturated GPLs in LID could lead to thinner, more permeable membranes^61^, whereas in non-LID animals, a higher proportion of saturated GPLs may support thicker, less permeable cell membranes. PUFA-containing GPLs, particularly omega-3 fatty acids like DHA, are natural ligands of peroxisome proliferator-activated receptors (PPARs). Their oxidation can activate PPARs, leading to peroxisome proliferation and increased plasmalogen PCs. PPARs are promising therapeutic targets for neurodegenerative diseases, including PD^63^ and LID^64^, due to their neuroprotective effects against oxidative damage and regulatory role in lipid metabolism, along with development, tissue differentiation, inflammation and mitochondrial function^63^.

Further, DHA-containing cellular membranes are known to increase permeability to small polar molecules^65^, and PUFA-containing GPLs are more enriched in axon tips than in cell bodies of cultured neurons, suggesting they may have a role in neurotransmitter release through modulation of membrane properties^61,66^. Our previous findings of elevated levels of L-DOPA and its metabolite 3-*O*-methyldopa in several grey matter brain regions in the same LID animals, along with increased dopamine and metabolite levels, support the hypothesis that cellular membrane permeability may differ significantly between LID and non-LID animals^35^ Such differences may contribute to the altered L-DOPA levels, potentially due to blood-brain barrier impairment or L-DOPA efflux in non-LID animals, leading to dyskinesia. Certain essential fatty acids, such as DHA, are derived from diet, and changes in dietary intake can affect brain levels and activity in pathways regulated by PUFAs. Blood-brain barrier impairment in LID animals may also dysregulate PUFA intake. Interestingly, DHA has been shown to reduce LID in animal PD models, suggesting its therapeutic potential in managing LID.

Thirdly, in previous analyses of MPTP vs. Ctrl animals using brain sections at -6 mm from the ac, we observed significant lipid changes, primarily in sulfatides. Long-chain non-hydroxylated sulfatides were depleted, whereas shorter long-chain non-hydroxylated sulfatides were elevated in basal ganglia brain regions, including the GPi, GPe and SNR^42^. In this study, certain sphingolipids, such as hydroxylated hexosylceramides and non-hydroxylated SMs, were also depleted in MPTP compared to Ctrl animals. Further, targeted analysis revealed a general trend of decreased hydroxylated SHexCers and increased non-hydroxylated SHexCers in both MPTP vs. Ctrl and LID vs. non-LID animals.

In this study, we also evaluated the ratios of hydroxylated (t) and non-hydroxylated (d) SHexCers in Ctrl, MPTP, non-LID and LID animals. The results revealed a clear pattern: t/d ratios of SHexCers were reduced in both MPTP compared to Ctrl animals and LID compared to non-LID animals. Interestingly, ratios of PUFA-containing SHexCers were elevated in non-LID compared to MPTP animals, whereas monounsaturated SHexCers did not show this pattern in the GPi and GPe, suggesting selective L-DOPA-induced alterations in PUFA-containing SHexCers.

A diverse range of sphingolipids has been identified in the nervous system as essential components of cellular membranes, and they play critical roles in maintaining brain function, particularly neurogenesis and synaptogenesis^4,67^. Sphingolipid biochemistry is increasingly recognized as a factor in neurodegenerative diseases^4^. Cerebrosides and SMs are typically more abundant in white matter than in grey matter, while glycosynapses, comprising hexosylceramides, galactosylceramides and sulfatides, contribute to long-term myelin stability^68^. We recently discovered distinct distributions of several hydroxylated and non-hydroxylated sulfatides with different acyl chain lengths within several brain regions. Hydroxylated sulfatides were predominantly distributed within grey matter areas, particularly within motor-related brain regions and temporal cortical areas in macaque brain tissue sections^42^.

We also reported depletion of several long-chain hydroxylated sulfatides in motor-related brain regions of MPTP-induced PD monkey brains, including the GPi and GPe, whereas some non-hydroxylated sulfatides were present at elevated levels in multiple brain regions, including the GPi and GPe^42^. In the present study, ratios of long-chain hydroxylated to non-hydroxylated sulfatides were significantly reduced in MPTP compared to Ctrl animals and in LID compared to non-LID animals. These results suggest that the hydroxylation mechanism may influence alterations of sulfatides in both experimental MPTP Parkinsonism and LID. The higher abundance of hydroxylated sulfatides in motor-related grey matter frequently forms satellite structures around neurons that wrap around nerve axons, potentially regulating the neuronal microenvironment^69^. An altered composition of hydroxylated and non-hydroxylated sulfatides may disrupt myelin stability or myelin sheath function around axons, impacting synaptic transmission.

The increased ratios of PUFA-containing SHexCers (but not monounsaturated) in non-LID compared to MPTP animals may reflect L-DOPA-induced changes of myelin structure in axonal regions of the GPi and GPe. Nevertheless, the observed depletion of long-chain hydroxylated SHexCers and HexCers may be linked to peroxisomal α-oxidation, a pathway involved in hydroxylated fatty acid degradation within sphingolipids. Enzymes in this pathway catalyse the one-carbon cleavage of hydroxylated fatty acids in the peroxisome, resulting in non-hydroxylated fatty acids that can be reused for sphingolipid synthesis in the endoplasmic reticulum^70,71^. These processes may underpin the altered composition of hydroxylated and non-hydroxylated sulfatide composition observed in PD and LID brains.

Although our findings provide detailed biochemical insights into lipid dysregulation in LID, future studies employing orthogonal methods or functional validation experiments would be valuable to further explore causality and evaluate the therapeutic potential of these lipid alterations. Furthermore, given that lipid measurements were conducted after symptom onset, our findings remain inherently correlative. While strongly supported by literature and observed biochemical correlations, the proposed mechanistic roles for these lipids in LID pathology should be considered hypothesis-generating rather than definitive.

In conclusion, we applied MALDI-MSI to conduct a comprehensive, region-specific lipidomics analysis of coronal primate brain tissue sections, capturing the complex lipid alterations associated with PD and LID. Our findings revealed significant, region-specific changes in plasmalogen PCs, GPLs and sphingolipids, including both hydroxylated and non-hydroxylated sulfatides, particularly in motor-related brain areas. The observed lipid alterations suggest peroxisomal dysfunction in LID, implicating these lipid species in oxidative stress susceptibility and membrane instability within key brain regions affected by PD and LID.

Importantly, our data highlighted specific correlations between lipid changes and clinical parameters, including LID severity scores, DAT binding and levels of L-DOPA and dopamine, indicating a potential mechanistic link between dysregulated lipid metabolism and the pathological, neurochemical and behavioural manifestations of LID. For instance, depletion of plasmalogen PCs and PUFA-containing GPLs in LID suggests a role in increased membrane fluidity and oxidative vulnerability, whereas the imbalance in hydroxylated and non-hydroxylated sulfatides points to potential disruptions in myelin integrity and neuronal signalling. These results offer new insights into the molecular mechanisms underlying LID and PD, underscoring the importance of lipid metabolism in disease progression and response to L-DOPA treatment. By mapping these lipidomic alterations in a non-human primate model closely aligned with human PD, this study establishes a foundation for further research into lipid-targeted therapies. Our findings pave the way for future studies to explore specific lipids as therapeutic targets or biomarkers for PD and LID, potentially informing the development of lipid-based strategies to mitigate dyskinesia and enhance treatment outcomes in PD.

## Methods

### Chemicals and reagents

All chemicals used in sample preparation were pro-analysis grade and obtained from Sigma-Aldrich, unless otherwise specified. Acetonitrile and water were purchased from VWR (Stockholm, Sweden).

### Ethical Statement

Animal experiments were carried out in accordance with the European Communities Council Directive of November 24, 1986 (86/609/EEC), revised in 2010 (2010/63/EU), regarding care of laboratory animals in an AAALAC-accredited facility following acceptance of the study design by the Institute of Lab Animal Science (Chinese Academy of Science, Beijing, China) IACUC for experiments conducted on non-human primates. An experienced and skilled veterinarian supervised the animals’ care and health throughout the experiments.

### Animal experiments

Experiments were performed on brain tissue samples from a previously published brain bank^35,39,44,45^. Female rhesus monkeys (Macaca mulatta: mean age = 5 ± 1 years, mean weight = 5.3 ± 0.8 kg, *n* = 29) were randomly assigned to different treatment groups. Control animals (*n* = 5) received daily saline injections, whereas the remainder received a daily dose of MPTP (0.2 mg/kg, i.v., Sigma, St. Louis, MO) to induce Parkinsonian symptoms, according to previously published protocols^41,72^. Following stabilization of the MPTP-induced symptoms, animals received either saline (*n* = 5) or L-DOPA (*n* = 12) injections twice daily for three months (20 mg/kg, p.o.). The animals were rated daily for Parkinsonian symptoms and dyskinesia using a PD clinical rating scale optimized for macaques^39,45,73^ and a dyskinesia disability scale^74^. The mean PD scores of the non-LID and LID groups before L-DOPA treatment were 10.2 and 9.3, respectively, with the minimum disability score being zero and the maximum being 25. Based on the dyskinesia scores, the animals were divided into a non-LID group (*n* = 6) comprising animals scoring zero and a LID group (*n* = 6) comprising animals scoring above zero (the mean score for these animals was 2.3 out of 4, indicating moderate dyskinesia). Animals were euthanized with an overdose of pentobarbital (150 mg/kg) one hour after the last L-DOPA dose. Brains were quickly removed after death, immediately frozen in dry-ice-cooled isopentane (at −45 °C), and then stored at −80 °C. The time between euthanasia and freezing of the brains was 10 min for all the animals. The two hemispheres were separated, with one stored at -80 °C until sectioning for MALDI-MSI analysis.

### Tissue processing

For MALDI-FTICR-MSI analysis, coronal brain tissue sections (−4 mm from the anterior commissure)^49^ from one hemisphere of all control, MPTP-treated, non-LID and LID animals were sectioned at a thickness of 12 μm in a cryostat (Leica CM3050S, Leica Microsystems, Wetzlar, Germany) at −20 °C. Sections were then thaw-mounted onto indium tin oxide-coated glass slides (Bruker Daltonics, Bremen, Germany) for MALDI-FTICR-MSI and stored at −80 °C prior to analysis.

### Design of MALDI-MSI experiments

Each control together with an MPTP sample or non-LID together with LID sample was analysed on direct consecutive days under the same experimental conditions. All pairs were randomly selected. Analysing coronal brain sections taken at −4 mm relative to the ac^49^ enabled investigation of motor-related brain areas important for PD pathogenesis, including the caudate (Cd) and putamen (Put), the precentral gyrus (PrG, where the primary motor cortex is located), and the internal and external segments of the globus pallidus (GPi/GPe).

### Sample preparation for MALDI-MSI

Sample preparation for MALDI-FTICR-MSI of lipids was performed as previously described^42^. Briefly, sections were desiccated at room temperature for 15 min before spray coating them with a norharmane matrix solution for dual polarity analysis of lipids^47^. Prior to matrix coating, the slide was scanned on a flatbed scanner (Epson Perfection V500, Nagano, Japan). The matrix solution was prepared by dissolving norharmane matrix powder in an 80% MeOH (7.5 mg/ml) solution in a glass vial, followed by brief sonication. An automated pneumatic sprayer (HTX TM-Sprayer, HTX-Technologies LLC, Chapel Hill, NC, USA) combined with an HPLC pump (AKTA FPLC P-905 pump, Amersham Pharmacia Biotech, Amersham, UK) was used to spray heated matrix solution over the tissue sections. The pump was kept running at 70 μL/min using 50% ACN as a pushing solvent at isocratic pressure before the experiments to ensure a stable flow of the solvent. The matrix solution was sprayed using the following instrumental parameters: solvent flow rate 70 μL/min; nitrogen pressure 6 psi; nozzle spray temperature 60 °C; 15 passes (all horizontal); nozzle head velocity 1200 mm/min; track spacing 2.0 mm.

### MALDI-FTICR-MSI analysis

All MALDI-MSI experiments for lipid imaging were performed in both negative and positive ionization modes on the same tissue sections using a MALDI-FTICR (Solarix XR 7T-2ω, Bruker Daltonics) mass spectrometer equipped with a Smartbeam II 2 kHz laser. The size of the laser was chosen to give a lateral resolution of 150 μm in both polarities, with an offset value of 75 μm to ensure no laser ablation overlaps when switching between polarities. The instrument was tuned for optimal detection of lipid molecules (*m*/*z* 200−2000) in both polarities using the quadrature phase detection (QPD) (2*ω*) mode. The transient length was 0.73 s, providing a mass resolution of about 106,000 at *m*/*z* 850 during the MSI analysis of lipids on brain tissue sections.

Time-of-flight (TOF) values were set at 0.8 and 1.0 ms for the positive and negative ion mode analyses, respectively, and the transfer optics frequency was kept at 4 MHz for both polarities. The quadrupole isolation *m*/*z* value (Q1 mass) was set at *m*/*z* 220.00 for both polarity modes. For both polarity modes, spectra were collected by summing 100 laser shots per pixel. Both methods were calibrated externally with red phosphorus over an appropriate mass range. Ion signals at *m/z* 885.549853 (monoisotopic peak of [PI(38:4)-H]^-^) and *m/z* 798.540963 (monoisotopic peak of [PC(34:1) + K]^+^) were used for internal calibration of the negative and positive polarity analyses, respectively. The laser power was optimized at the start of each analysis and then held constant during the MALDI-MSI experiment.

### Data processing and analysis

MALDI-FTICR-MSI data in dual polarity from five pairs of control and MPTP-lesioned samples, and six pairs of non-LID and LID samples were initially visualized in FlexImaging (v.5.0, Bruker Daltonics). For further analysis, all data were imported to SCiLS Lab (version 2023b Pro, Bruker Daltonics) and combined for negative and positive polarity data in separate files, in which brain regions were annotated according to a stereotaxic template atlas of the macaque brain^49^ aided by the correlation of lipid ion distributions with white and grey matter structures within the brain tissue sections. This spatial alignment was previously validated using Luxol fast blue and Cresyl Echt violet staining^42^.

Several discrete brain regions, the Cd and Put, PrG and GPi/GPe, postcentral gyrus (PoG), temporal gyrus (TG), i.e., the combined areas of the superior temporal gyrus (STG), middle temporal gyrus (MTG) and inferior temporal gyrus (ITG), along with the insula (Ins), claustrum (Cl), anterior cingulate gyrus (ACgG), temporal white matter (tw) and cerebral white matter (cw), were chosen for analysis. The sliding window function of SCiLS Lab was used to extract root mean square (RMS) normalized average peak area values for 2429 peaks in the negative ion mode and 2450 peaks in the positive ion mode separately from dual polarity MALDI-FTICR-MSI data. Corresponding peak area values of all *m*/*z* features were used to perform two-tailed *t*-tests and construct volcano plots between Ctrl vs. MPTP and non-LID vs. LID samples. Log10 fold changes (FC) were calculated based on the ratio of the average abundance of a *m*/*z* feature in MPTP vs. Ctrl sections and LID vs. non-LID sections. For the identified lipid species from volcano plots, the significance of the changes between Ctrl vs. MPTP and non-LID vs. LID were evaluated using Student’s *t*-test.

Before hypothesis testing, peak area values were log10-transformed, and data normality was assessed using the Shapiro–Wilk test. Statistical analysis was conducted in an untargeted manner, encompassing 4879 *m*/*z* features acquired in dual polarity mode. However, not all *m*/*z* features represent unique lipid species, as a single lipid can generate multiple signals due to isotopes, adducts, dimers, harmonic peaks, and Gibbs artifacts, resulting in substantial feature redundancy and non-independence.

Standard multiple testing corrections, such as Bonferroni or FDR, assume statistical independence across comparisons. In our dataset, this assumption does not hold and applying such corrections would lead to overcorrection and a heightened risk of false negatives, thereby obscuring biologically meaningful lipid alterations. Therefore, we focused on targeted two-group comparisons (MPTP vs. Ctrl and non-LID vs. LID) without correcting for multiple comparisons, aiming to identify consistent and biologically interpretable patterns of lipid changes across anatomically defined brain regions.

Inter-animal variability, calculated as the coefficient of variation (%CV) based on the summed intensities of all detected ions across analysed regions, was 5.28% in negative ion mode and 5.57% in positive ion mode. Heat maps were generated to evaluate trends in changes in the abundance of lipids using *z*-scores, which were calculated using the equation *z* = (*x*−*μ*)/*σ*, where *x* is the mean lipid abundance of biological replicates in a group, *μ* is the mean peptide abundance in samples from all animals and *σ* is the standard deviation of all samples. Pearson’s correlation analysis was performed between the levels of lipids and LID scores, DAT scores from the Cd and Put or levels of L-DOPA and dopamine. Before the Pearson correlation analysis, levels of lipids and L-DOPA and dopamine were log10 transformed. For visualization of the results, heat maps colour-coded according to Pearson’s correlation coefficients were constructed.

### Identification of lipids

An untargeted mass list including 4879 *m*/*z* values in dual polarity was used for multiple *t*-test analysis, and *m/z* values showing *p* ≤ 0.05 were selected for identification (Supplementary Table 1) by conducting a search against the LIPID MAPS database with a 0.01*m/z* tolerance for both the negative and positive polarities, including all ion types. No deisotoping of the spectra was applied prior to lipid assignments.

MALDI-tandem MS (MS/MS) was performed on tissues by collecting spectra from brain regions where the target ion was abundant. The resulting product ions were compared to product ion spectra of standards in the LIPID MAPS database (Nature Lipidomics Gateway, www.lipidmaps.org) and/or previously published data^75,76^. In cases where sodium and/or potassium adduct ions of the same lipid species were identified, the brain tissue distribution of the adducts and [M + H]^+^ or [M-H]^−^ ions were compared (Supplementary Figs. 9–21). Lipids were assigned based on the mass accuracy in instances where the peak intensity was insufficient for MS/MS analysis of the tissue. Fatty acid chain or long-chain base specific information of some lipids was obtained by MS/MS (see Supplementary Table 2). For a detailed explanation of lipid annotations, refer to Supplementary Table 2, 3.

Several hydroxylated and non-hydroxylated sulfatide species (Fig. 6 and Supplementary Figs. 5, 7 and 8) were annotated from our previous study examining sulfatides in MPTP primate coronal brain tissue sections^42^.

## Supplementary information

240713-LID-Lipids-Suppl-Info

Supplementary-Data

## Data Availability

The source data, including the average peak area of the lipids in all animals, is available online in the Supplementary Data. The MALDI-MSI datasets used and/or analysed during the current study are available from the corresponding author on request.
