# Supplementary material for: Brain-region-specific lipid dysregulation in L-DOPA-induced dyskinesia in a primate model of Parkinson’s disease
Source: NPJ Parkinsons Dis. 2025 Aug 23;11:258. doi: 10.1038/s41531-025-01109-6 (PMC12374971; doi:10.1038/s41531-025-01109-6)
Supplement: Supplementary file 1 — 240713-LID-Lipids-Suppl-Info [file 41531_2025_1109_MOESM1_ESM.pdf]

SUPPLEMENTARY INFORMATION

Brain-Region-Specific Lipid Dysregulation in L-DOPA-Induced Dyskinesia in a Primate Model of Parkinson's Disease

Ibrahim Kaya[1], Theodosia Vallianatou[1], Anna Nilsson[1], Patrik Bjärterot[1], Reza Shariatgorji[1], Per Svenningsson[2], Erwan Bezard[3,4,5], Per E. Andrén [1]*

1. Department of Pharmaceutical Biosciences, Spatial Mass Spectrometry, Science for Life Laboratory, Uppsala University, Uppsala, Sweden.
2. Section of Neurology, Department of Clinical Neuroscience, Karolinska Institutet, Stockholm, Sweden.
3. Université de Bordeaux, Institut des Maladies Neurodégénératives, Bordeaux, France.
4. CNRS, Institut des Maladies Neurodégénératives, Bordeaux, France.
5. Motac Neuroscience, Bordeaux, France.

Corresponding Author

Per E. Andrén, Department of Pharmaceutical Biosciences, Spatial Mass Spectrometry, Uppsala University, Uppsala, Sweden

Email. per.andren@ uu.se

Phone. +46-70 167 9334

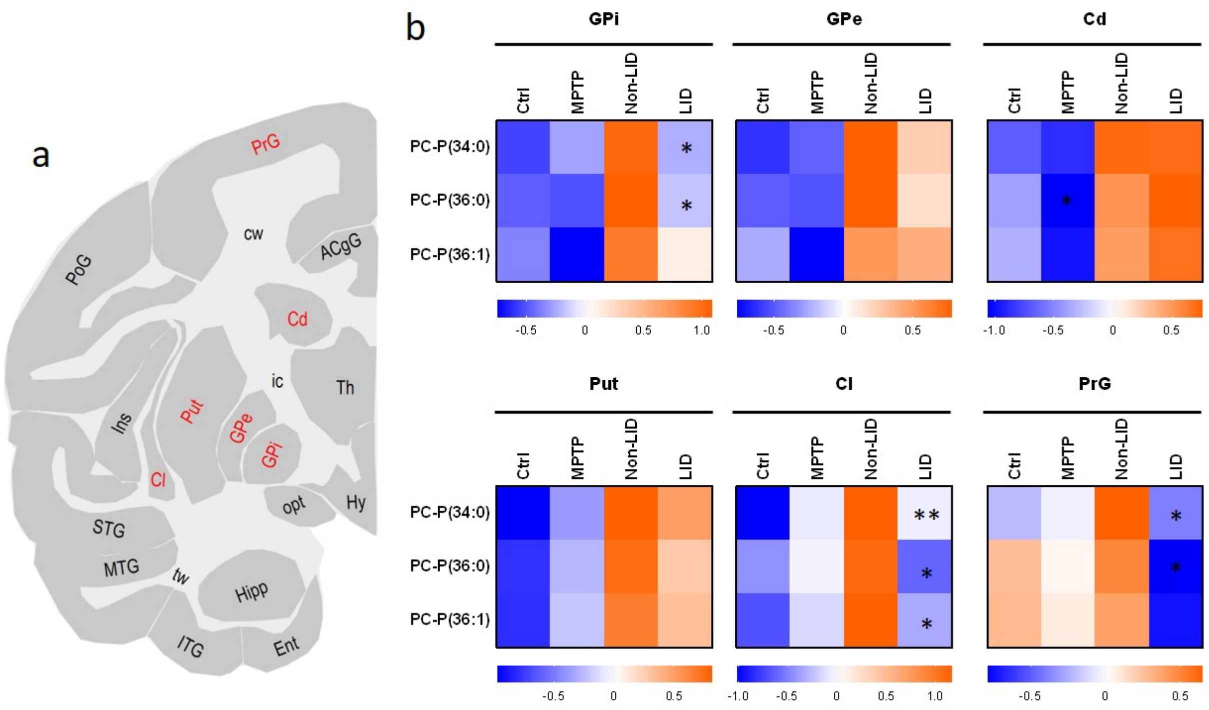

Supplementary Fig 1. Brain-region-specific changes in plasmalogen PCs in Ctrl, MPTP, non-LID and LID animals. (a) Schematic of a coronal non-human primate brain tissue section at -4 mm from the ac depicting different brain regions, with those evaluated labeled in red. **(b)** Heat maps showing *z*-scores of plasmalogen PCs in the GPi, GPe, Cd, Put, Cl and PrG. Changes were evaluated between Ctrl vs. MPTP and non-LID vs. LID using Student's *t*-test. Asterisks indicate significance: **P*<0.05; ***P*<0.01; ****P*<0.001. Asterisks in the MPTP and LID columns show results of statistical analysis between Ctrl vs. MPTP and LID vs. non-LID groups, respectively. Abbreviations: PoG: postcentral gyrus; PrG: precentral gyrus; STG: superior temporal gyrus; ACgG: anterior cingulate gyrus; MTG: middle temporal gyrus; ITG: inferior temporal gyrus; Ent: entorhinal area; Hipp: hippocampus; Cd: caudate nucleus; Ins: insula; opt: optical tract; Cl: claustrum; Put: putamen; GPe/GPi: globus pallidus externa/interna; Hy: hypothalamus; Th: thalamus ic: internal capsule; tw: temporal white matter; cw: cerebral white matter.

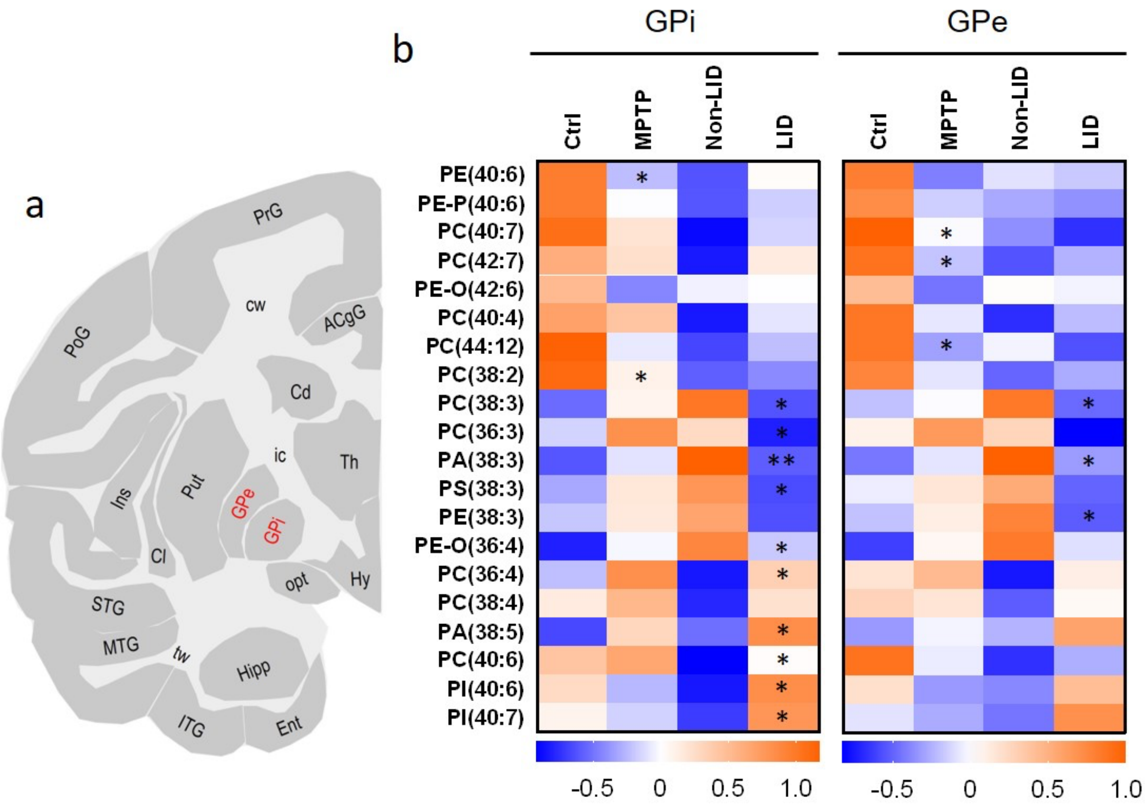

Supplementary Fig 2. Brain-region-specific changes in PUFA-containing GPLs in the GPi and GPe of Ctrl, MPTP, non-LID and LID animals. (a) Schematic of a coronal non-human primate brain tissue section at -4 mm from the ac depicting different brain regions, with those evaluated labeled in red. **(b)** Heat maps showing *z*-scores of PUFA-containing GPLs in the GPi and GPe. Changes were evaluated between Ctrl vs. MPTP and non-LID vs. LID using Student's *t*-test. Asterisks indicate significance: **P*<0.05; ***P*<0.01; ****P*<0.001. Asterisks in the MPTP and LID columns show results of statistical analysis between Ctrl vs. MPTP and LID vs. non-LID groups, respectively. Abbreviations: PoG: postcentral gyrus; PrG: precentral gyrus; STG: superior temporal gyrus; ACgG: anterior cingulate gyrus; MTG: middle temporal gyrus; ITG: inferior temporal gyrus; Ent: entorhinal area; Hipp: hippocampus; Cd: caudate nucleus; Ins: insula; opt: optical tract; Cl: claustrum; Put: putamen; GPe/GPi: globus pallidus externa/interna; Hy: hypothalamus; Th: thalamus ic: internal capsule; tw: temporal white matter; cw: cerebral white matter.

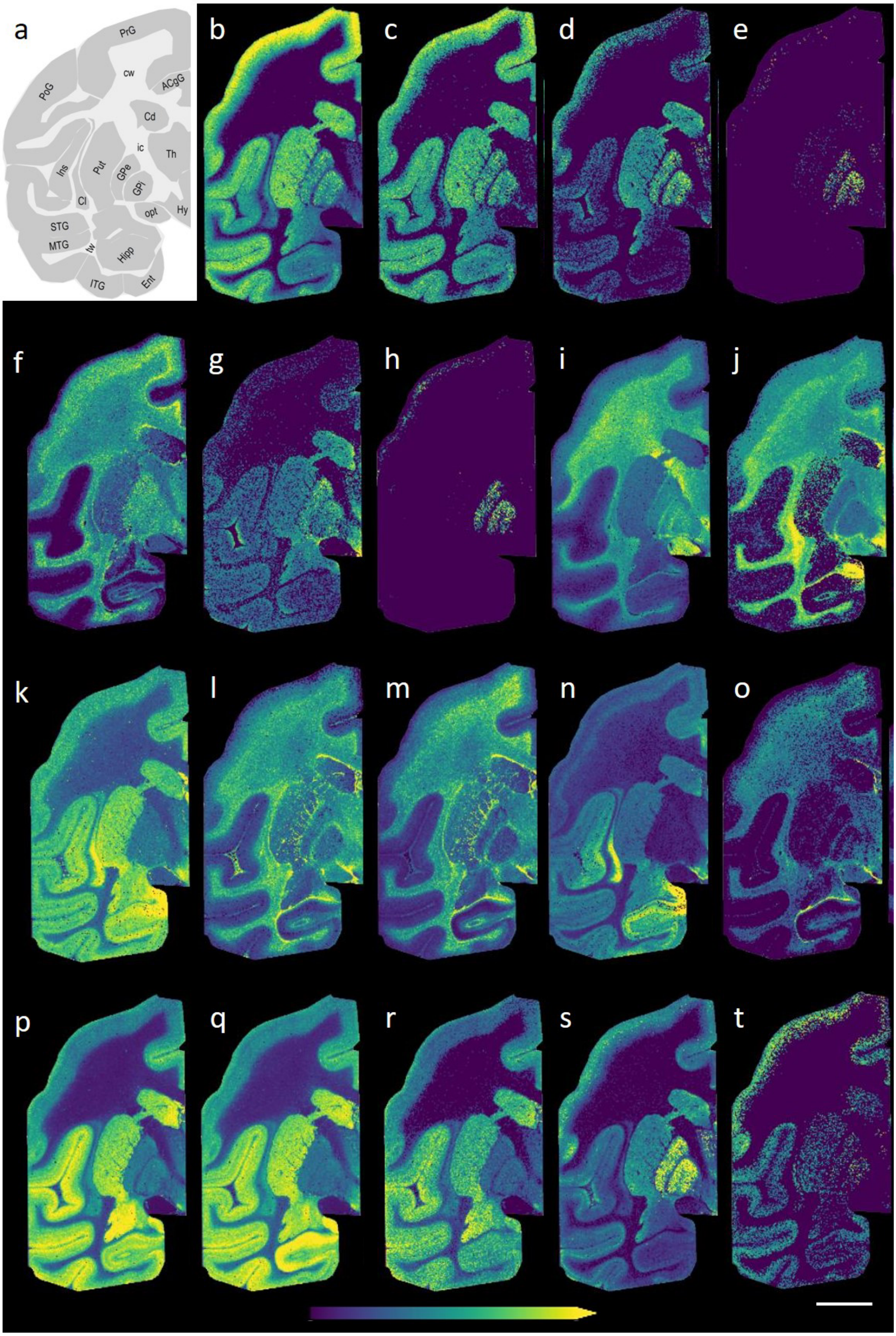

a
PrG
cw
ACgG
PoG
Cd
ic
Th
Ins
Put
GPe
GPi
Cl
opt
Hy
STG
MTG
tw
Hipp
ITG
Ent
b
c
d
e
f
g
h
i
j
k
l
m
n
o
p
q
r
s
t

Supplementary Fig 3. MALDI-FTICR-MSI data showing lateral ion distributions of PUFA-containing GPLs in non-human primate brain tissue sections. (a) Schematic of a coronal non-human primate brain tissue section at -4 mm from the ac depicting different brain regions. Ion images visualized at 150 µm lateral resolution in dual polarity mode of **(b)** PE(40:6) ($[M+K]^+$ ion), **(c)** PE-P(40:6) ($[M+K]^+$ ion), **(d)** PC(40:7) ($[M+Na]^+$ ion), **(e)** PC(42:7) ($[M+K]^+$ ion), **(f)** PE-O(42:6) ($[M-H]^-$ ion), **(g)** PC(40:4) ($[M+Na]^+$ ion), **(h)** PC(44:12) ($[M+K]^+$ ion), **(i)** PC(38:2) ($[M+K]^+$ ion), **(j)** PC(38:3) ($[M+K]^+$ ion), **(k)** PC(36:3) ($[M+K]^+$ ion), **(l)** PA(38:3) ($[M-H]^-$ ion), **(m)** PS(38:3) ($[M-H]^-$ ion), **(n)** PE(38:3) ($[M-H]^-$ ion), **(o)** PE-O(36:4) ($[M-H]^-$ ion), **(p)** PC(36:4) ($[M+K]^+$ ion), **(q)** PC(38:4) ($[M+K]^+$ ion), **(r)** PA(38:5) ($[M+K]^+$ ion), **(s)** PC(40:6) ($[M+Na]^+$ ion), and **(t)** PI(40:7) ($[M-H]^-$ ion). All ion distribution images are scaled to the maximum intensity of each individual ion and are RMS-normalized. Abbreviations: PoG: postcentral gyrus; PrG: precentral gyrus; STG: superior temporal gyrus; MTG: middle temporal gyrus; ITG: inferior temporal gyrus; ACgG: anterior cingulate gyrus; Ent: entorhinal area; Hipp: hippocampus; Cd: caudate nucleus; Ins: insula; opt: optical tract; Cl: claustrum; Put: putamen; GPe/GPi: globus pallidus externa/interna; Hy: hypothalamus; Th: thalamus ic: internal capsule; tw: temporal white matter; cw: cerebral white matter.

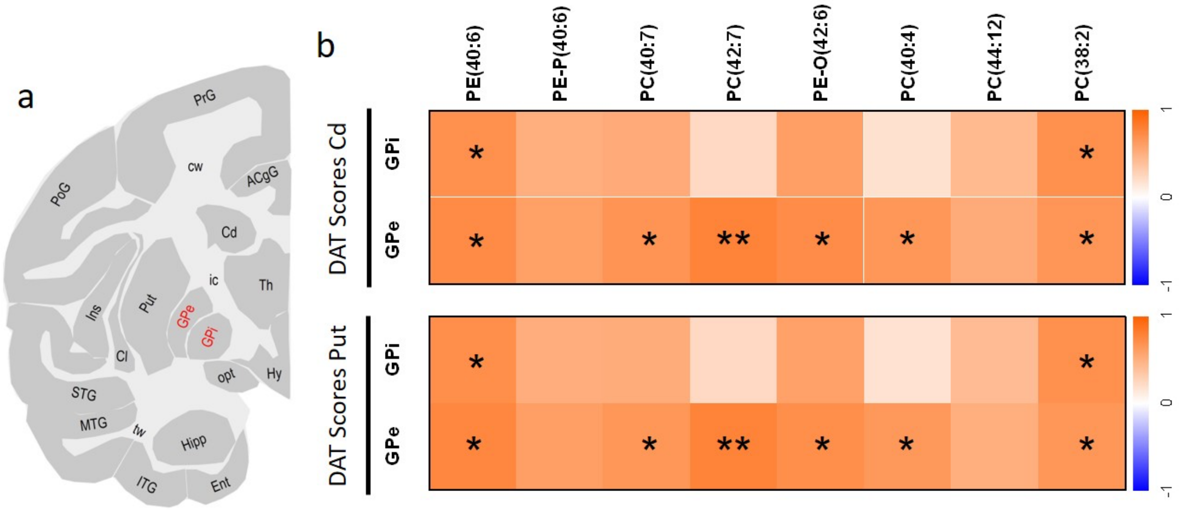

Supplementary Fig 4. Relationship between DAT binding scores from Cd and Put brain regions and PUFA-containing GPLs showing changes between MPTP and Ctrl animals. (a) Schematic of a coronal non-human primate brain tissue section at -4 mm from the ac depicting different brain regions, with those evaluated labeled in red. **(b)** Results of Pearson's correlation analysis between PUFA-containing GPL lipid levels in the GPi and GPe and DAT binding scores from the Cd and Put. Heat maps are color-coded according to Pearson's correlation coefficients: orange and blue colors indicate positive and negative correlations, respectively. Asterisks indicate significant correlations: *$P<0.05$; **$P<0.01$. Abbreviations: PoG: postcentral gyrus; PrG: precentral gyrus; STG: superior temporal gyrus; ACgG: anterior cingulate gyrus; MTG: middle temporal gyrus; ITG: inferior temporal gyrus; Ent: entorhinal area; Hipp: hippocampus; Cd: caudate nucleus; Ins: insula; opt: optical tract; Cl: claustrum; Put: putamen; GPe/GPi: globus pallidus externa/interna; Hy: hypothalamus; Th: thalamus ic: internal capsule; tw: temporal white matter; cw: cerebral white matter.

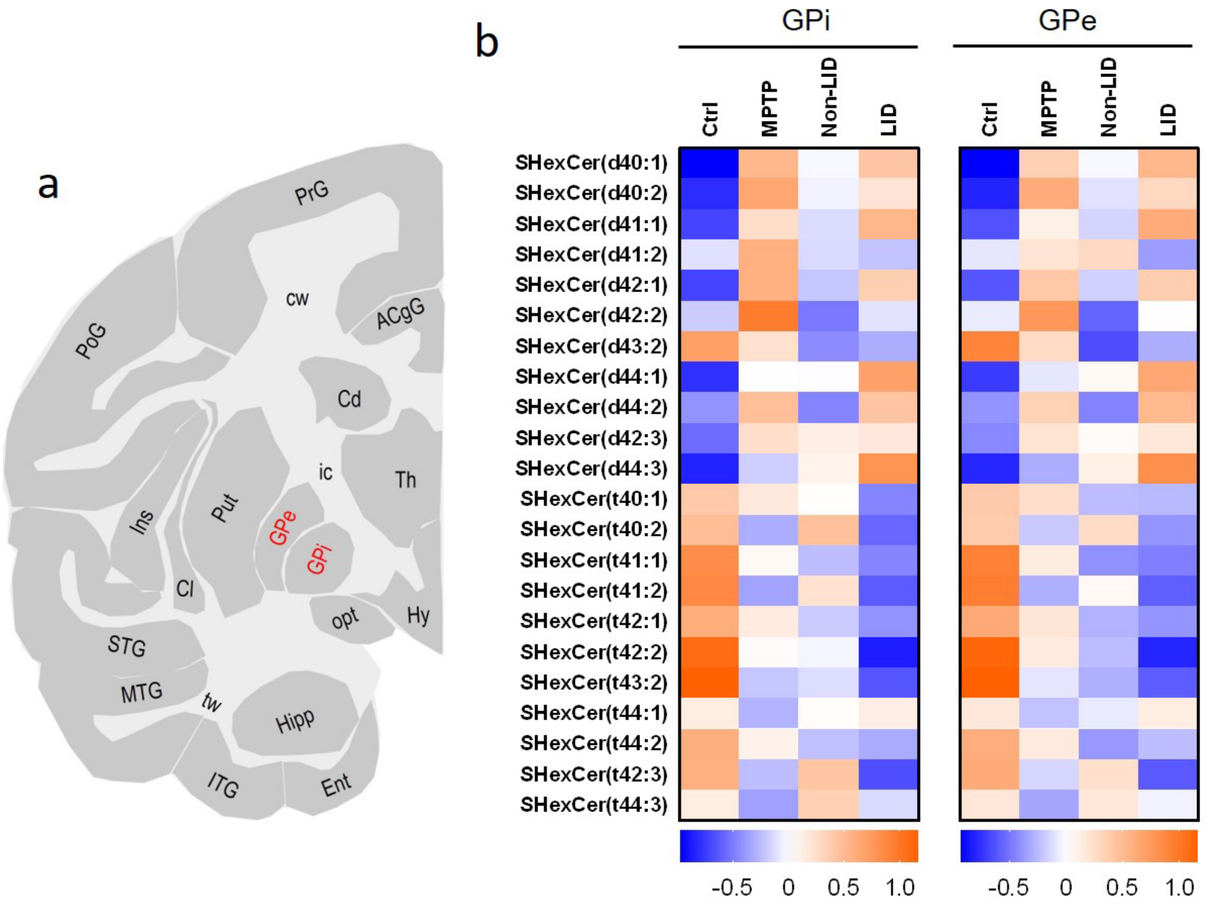

Supplementary Fig 5. Brain-region-specific changes in hydroxylated (t) and non-hydroxylated (d) SHexCer lipid species in the GPi and GPe of Ctrl, MPTP, non-LID and LID animals. a) Schematic of a coronal non-human primate brain tissue section at -4 mm from the ac depicting different brain regions, with those evaluated labeled in red. b) Heat maps showing the abundances with *z*-scores of hydroxylated (t) and non-hydroxylated (d) SHexCer lipids in the GPi and GPe brain regions in Ctrl, MPTP, non-LID and LID animals. Abbreviations: PoG: postcentral gyrus; PrG: precentral gyrus; STG: superior temporal gyrus; ACgG: anterior cingulate gyrus; MTG: middle temporal gyrus; ITG: inferior temporal gyrus; Ent: entorhinal area; Hipp: hippocampus; Cd: caudate nucleus; Ins: insula; opt: optical tract; Cl: claustrum; Put: putamen; GPe/GPi: globus pallidus externa/interna; Hy: hypothalamus; Th: thalamus ic: internal capsule; tw: temporal white matter; cw: cerebral white matter.

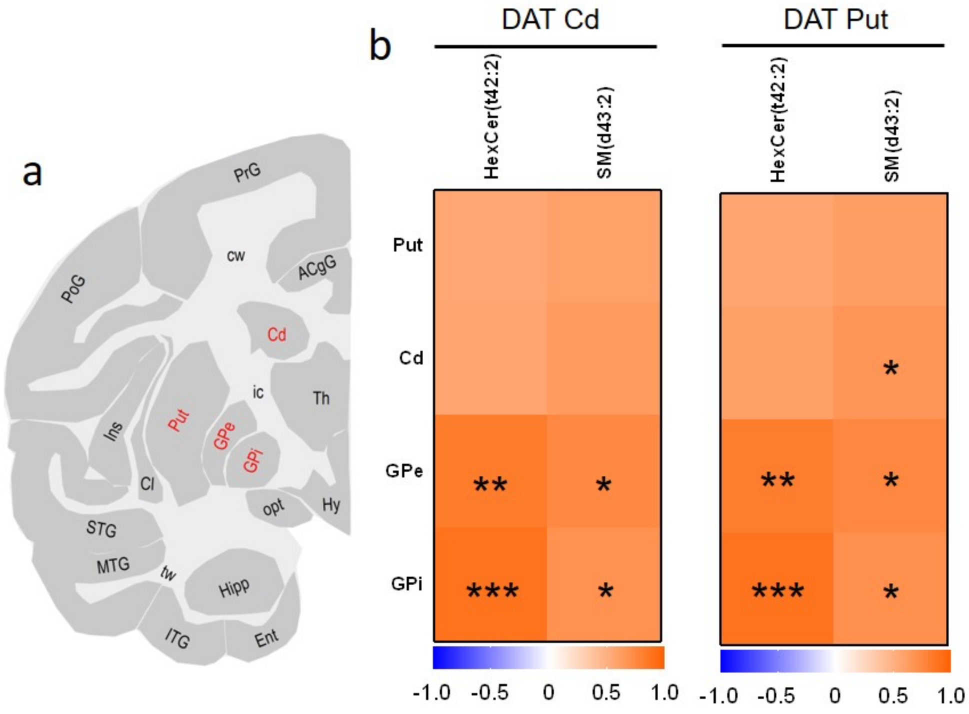

Supplementary Fig 6. Relationship between DAT binding scores from Cd and Put brain regions and sphingolipids showing changes between MPTP and Ctrl animals. a) Schematic of a coronal non-human primate brain tissue section at -4 mm from the ac depicting different brain regions, with those evaluated labeled in red. b) Results of Pearson's correlation analysis between HexCer(t42:2) and SM(d43:2) lipid levels in the GPi and GPe, and DAT binding scores from the Cd and Put. Heat maps are color-coded according to Pearson's correlation coefficients: orange and blue colors indicate positive and negative correlations, respectively. Asterisks indicate significant correlations: *P<0.05, **P<0.01, ***P<0.001. Abbreviations: PoG: postcentral gyrus; PrG: precentral gyrus; STG: superior temporal gyrus; ACgG: anterior cingulate gyrus; MTG: middle temporal gyrus; ITG: inferior temporal gyrus; Ent: entorhinal area; Hipp: hippocampus; Cd: caudate nucleus; Ins: insula; opt: optical tract; Cl: claustrum; Put: putamen; GPe/GPi: globus pallidus externa/interna; Hy: hypothalamus; Th: thalamus ic: internal capsule; tw: temporal white matter; cw: cerebral white matter.

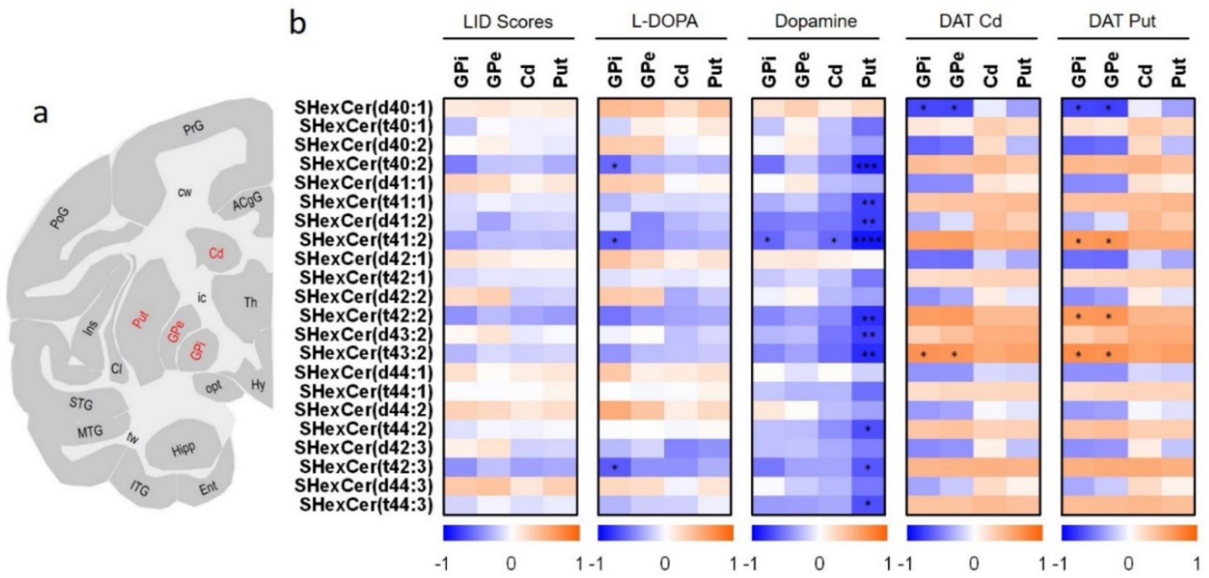

Supplementary Fig 7. Relationship between LID scores, L-DOPA levels, dopamine levels or DAT binding scores, and levels of hydroxylated (t) and non-hydroxylated (d) SHexCers lipid species. **(a)** Schematic of a coronal non-human primate brain tissue section at -4 mm from the ac depicting different brain regions, with those evaluated labeled in red. **(b)** Results of Pearson's correlation analysis of levels of hydroxylated (t) and non-hydroxylated (d) SHexCers in GPi, GPe, Cd and Put basal ganglia brain regions compared to LID scores of the animals, L-DOPA and dopamine levels obtained from the same regions of consecutive brain tissue sections and DAT binding scores obtained from the Cd and Put. Heat maps are color-coded according to Pearson's correlation coefficients: orange and blue colors indicate positive and negative correlations, respectively. Asterisks indicate significant correlations: **P*<0.05; ***P*<0.01, ****P*<0.001, *****P*<0.0001. Abbreviations: PoG: postcentral gyrus; PrG: precentral gyrus; STG: superior temporal gyrus; ACgG: anterior cingulate gyrus; MTG: middle temporal gyrus; ITG: inferior temporal gyrus; Ent: entorhinal area; Hipp: hippocampus; Cd: caudate nucleus; Ins: insula; opt: optical tract; Cl: claustrum; Put: putamen; GPe/GPi: globus pallidus externa/interna; Hy: hypothalamus; Th: thalamus ic: internal capsule; tw: temporal white matter; cw: cerebral white matter.

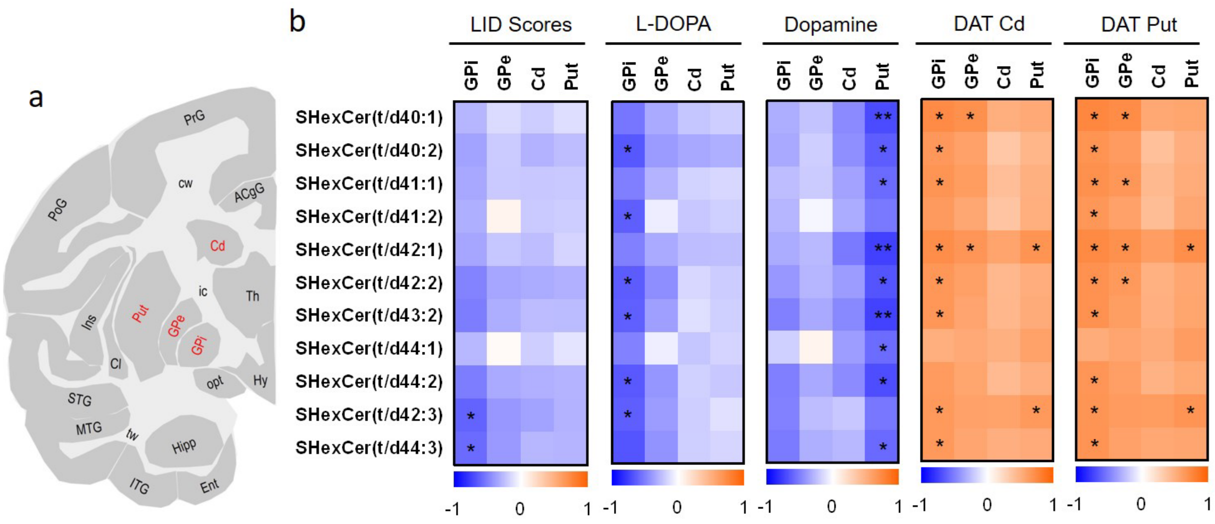

Supplementary Fig 8. Relationship between LID scores, L-DOPA levels, dopamine levels or DAT binding scores and ratios of hydroxylated (t) to non-hydroxylated (d) SHexCers lipid species. **(a)** Schematic of a coronal non-human primate brain tissue section at -4 mm from the ac depicting different brain regions, with those evaluated labeled in red. **(b)** Results of Pearson's correlation analysis of ratios of hydroxylated (t) to non-hydroxylated (d) SHexCers in the GPi, GPe, Cd and Put compared to LID scores of the animals, L-DOPA and dopamine levels obtained from the same regions in consecutive brain tissue sections and DAT binding scores obtained from the Cd and Put. Heat maps are color-coded according to Pearson's correlation coefficients: orange and blue colors indicate positive and negative correlations, respectively. Asterisks indicate significant correlations: *P<0.05; **P<0.01, ***P<0.001. Abbreviations: PoG: postcentral gyrus; PrG: precentral gyrus; STG: superior temporal gyrus; ACgG: anterior cingulate gyrus; MTG: middle temporal gyrus; ITG: inferior temporal gyrus; Ent: entorhinal area; Hipp: hippocampus; Cd: caudate nucleus; Ins: insula; opt: optical tract; Cl: claustrum; Put: putamen; GPe/GPi: globus pallidus externa/interna; Hy: hypothalamus; Th: thalamus ic: internal capsule; tw: temporal white matter; cw: cerebral white matter.

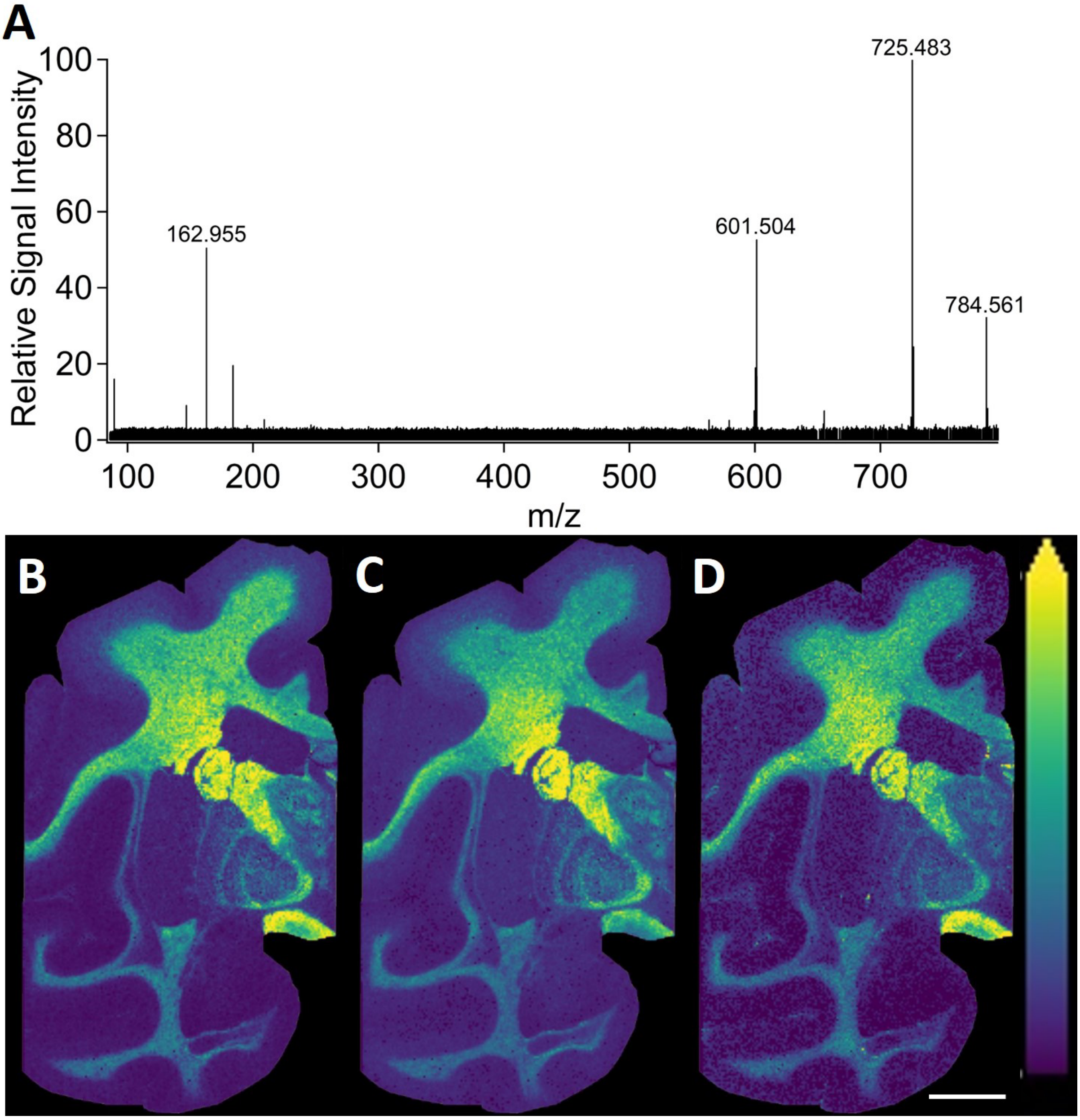

Supplementary Fig 9. MALDI-MS/MS spectra obtained from a coronal macaque brain tissue section using MALDI-CID-FTICR MS/MS. (a) Precursor ion at *m/z* 784.561. Product ions supporting the assignment of [PC-P(34:0)+K]$^+$ are at *m/z* 725.483 (loss of trimethylamine), *m/z* 601.504 (loss of phosphocholine head group) and *m/z* 162.955 (potassiated cyclophosphane). Due to the isolation width of 1 *m/z* unit, additional isobaric and isomeric lipids may have also fragmented, giving rise to additional product ions. MALDI-FTICR-MSI data revealing similar ion distribution images of **(b)** [PC-P(34:0)+H]$^+$, **(c)** [PC-P(34:0)+Na]$^+$, and **(d)** [PC-P(34:0)+K]$^+$ in a control coronal macaque brain tissue section. All images are RMS-normalized and scaled to the maximum intensity of each ion. Lateral resolution: 150 µm; scale bar: 7 mm.

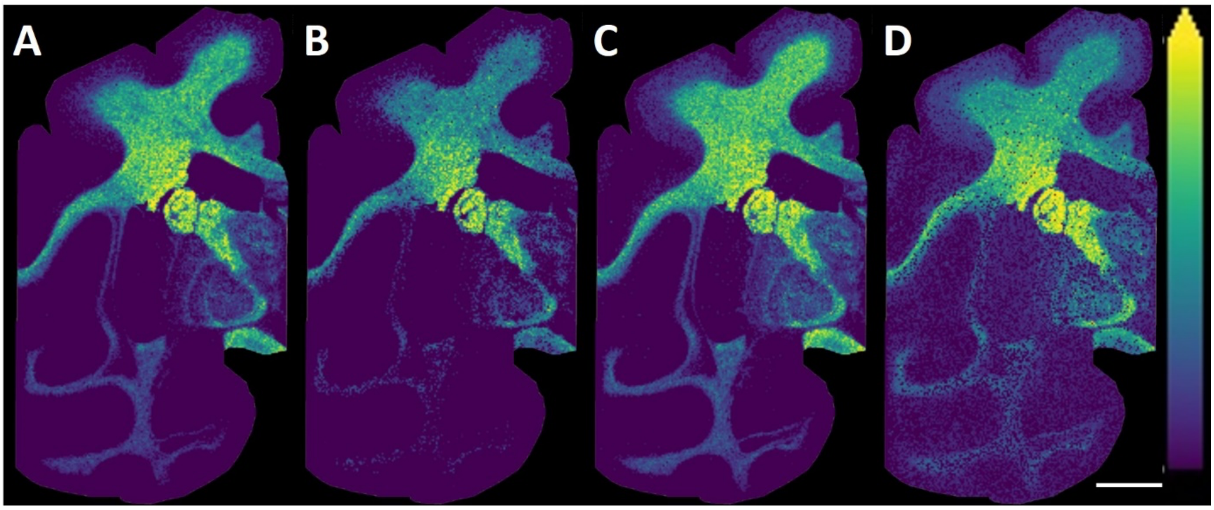

Supplementary Fig. 10. Similar distributions of plasmalogen PC ion types revealed by MALDI-FTICR-MSI. Ion images show the distributions of **(a)** $[PC\text{-}P(36:0)+H]^+$, **(b)** $[PC\text{-}P(36:0)+K]^+$, **(c)** $[PC\text{-}P(36:1)+H]^+$, and **(d)** $[PC\text{-}P(36:1)+K]^+$ in a control coronal macaque brain tissue section. All images are RMS-normalized and scaled to the maximum intensity of each ion. Lateral resolution: 150 μm; scale bar: 7 mm.

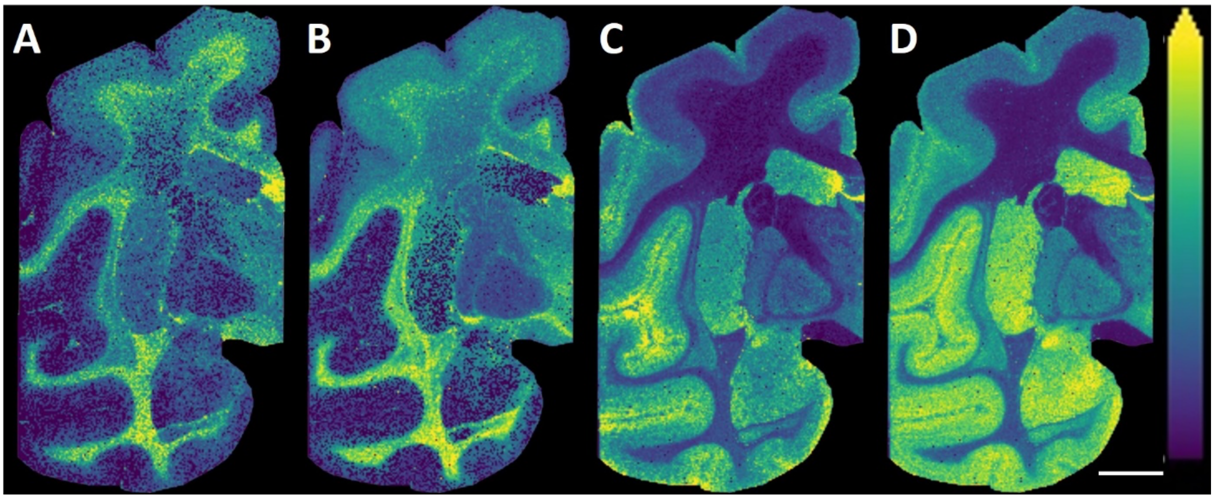

Supplementary Fig. 11. Similar distributions of PC ion types revealed by MALDI-FTICR-MSI. Ion images show the distributions of **(a)** $[PC(38:3)+H]^+$, **(b)** $[PC(38:3)+K]^+$, **(c)** $[PC(36:4)+Na]^+$, and **(d)** $[PC(36:4)+K]^+$ in a control coronal macaque brain tissue section. All images are RMS-normalized and scaled to the maximum intensity of each ion. Lateral resolution: 150 μm; scale bar: 7 mm.

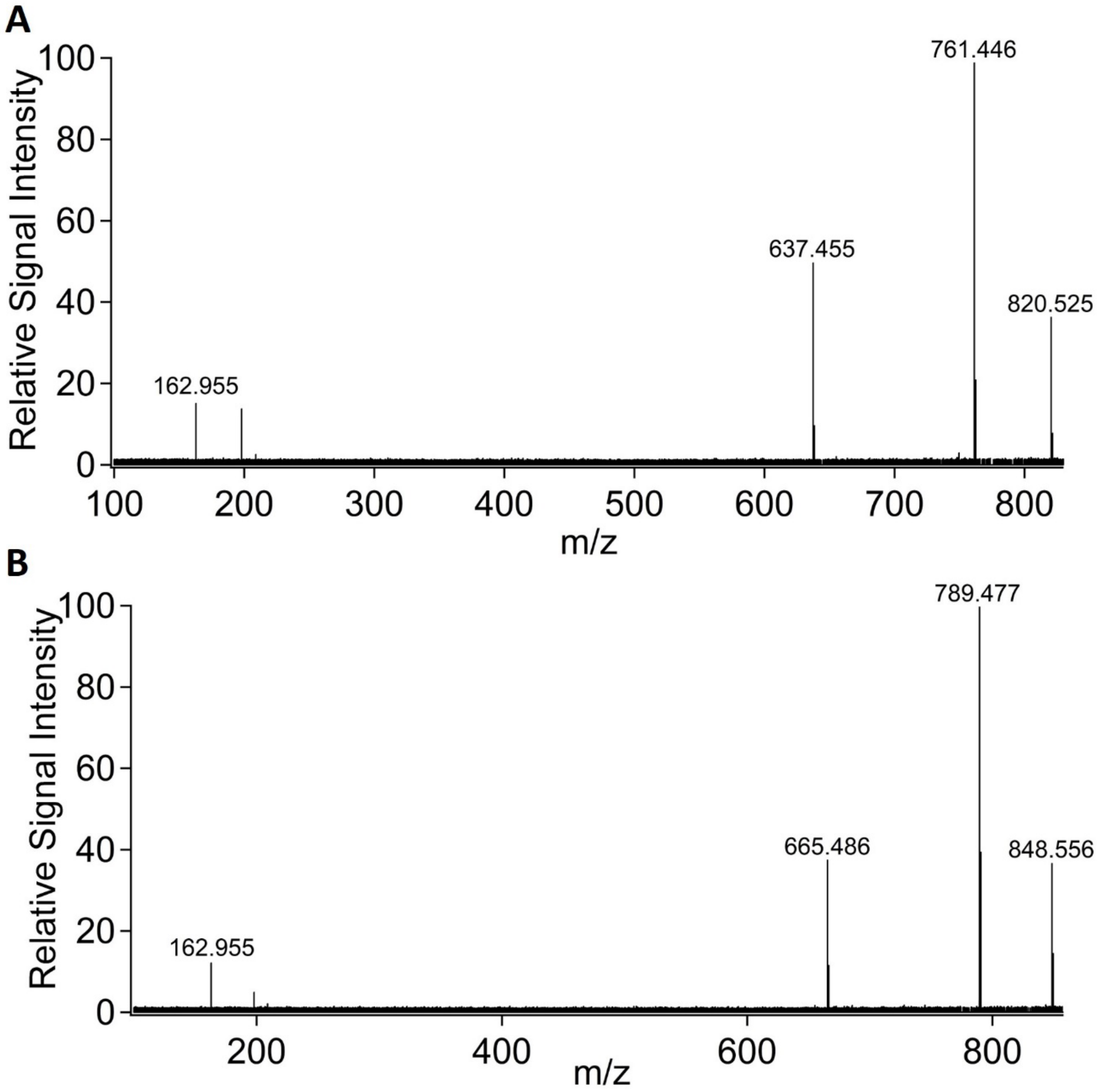

Supplementary Fig 12. MALDI-MS/MS spectra obtained from a coronal macaque brain tissue section using MALDI-CID-FTICR MS/MS. Precursor ions at **(a)** *m/z* 820.525, and **(b)** *m/z* 848.556. Product ions supporting the assignment of $[PC (36:4)+K]^+$ are at *m/z* 761.446 (loss of trimethylamine), *m/z* 637.455 (loss of phosphocholine head group) and *m/z* 162.955 (potassiated cyclophosphane). Product ions supporting the assignment of $[PC (38:4)+K]^+$ are at *m/z* 789.477 (loss of trimethylamine), *m/z* 665.486 (loss of phosphocholine head group) and *m/z* 162.955 (potassiated cyclophosphane). Due to the isolation width of 1 *m/z* unit, additional isobaric and isomeric lipids may have also fragmented, giving rise to additional product ions.

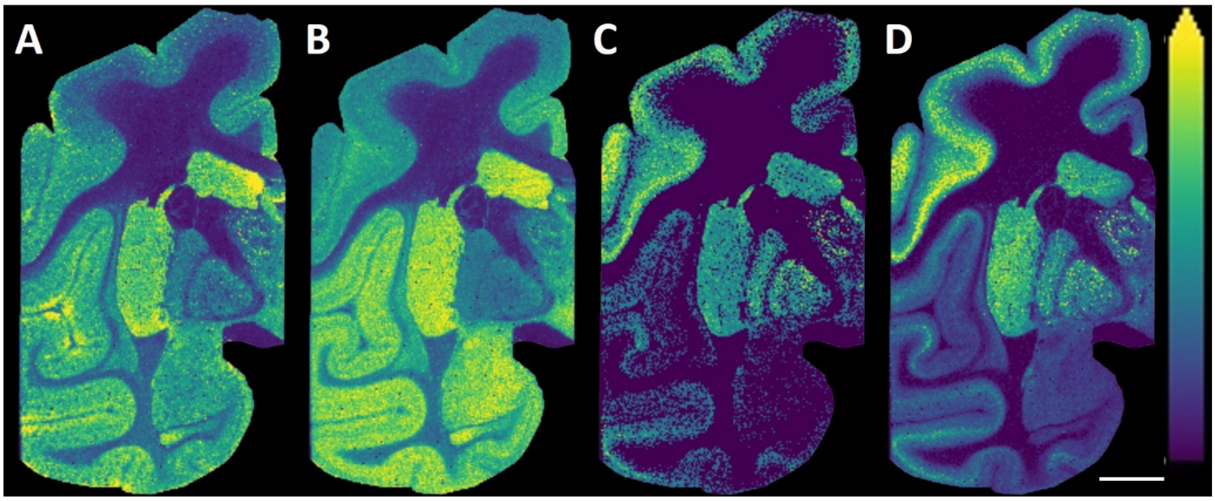

Supplementary Fig. 13. Similar distributions of PC ion types revealed by MALDI-FTICR-MSI. Ion images show the distributions of **(a)** $[PC(38:4)+Na]^+$, **(b)** $[PC(38:4)+K]^+$, **(c)** $[PC(40:7)+Na]^+$, and **(d)** $[PC(40:7)+K]^+$ in a control coronal macaque brain tissue section. All images are RMS-normalized and scaled to the maximum intensity of each ion. Lateral resolution: 150 µm; scale bar: 7 mm.

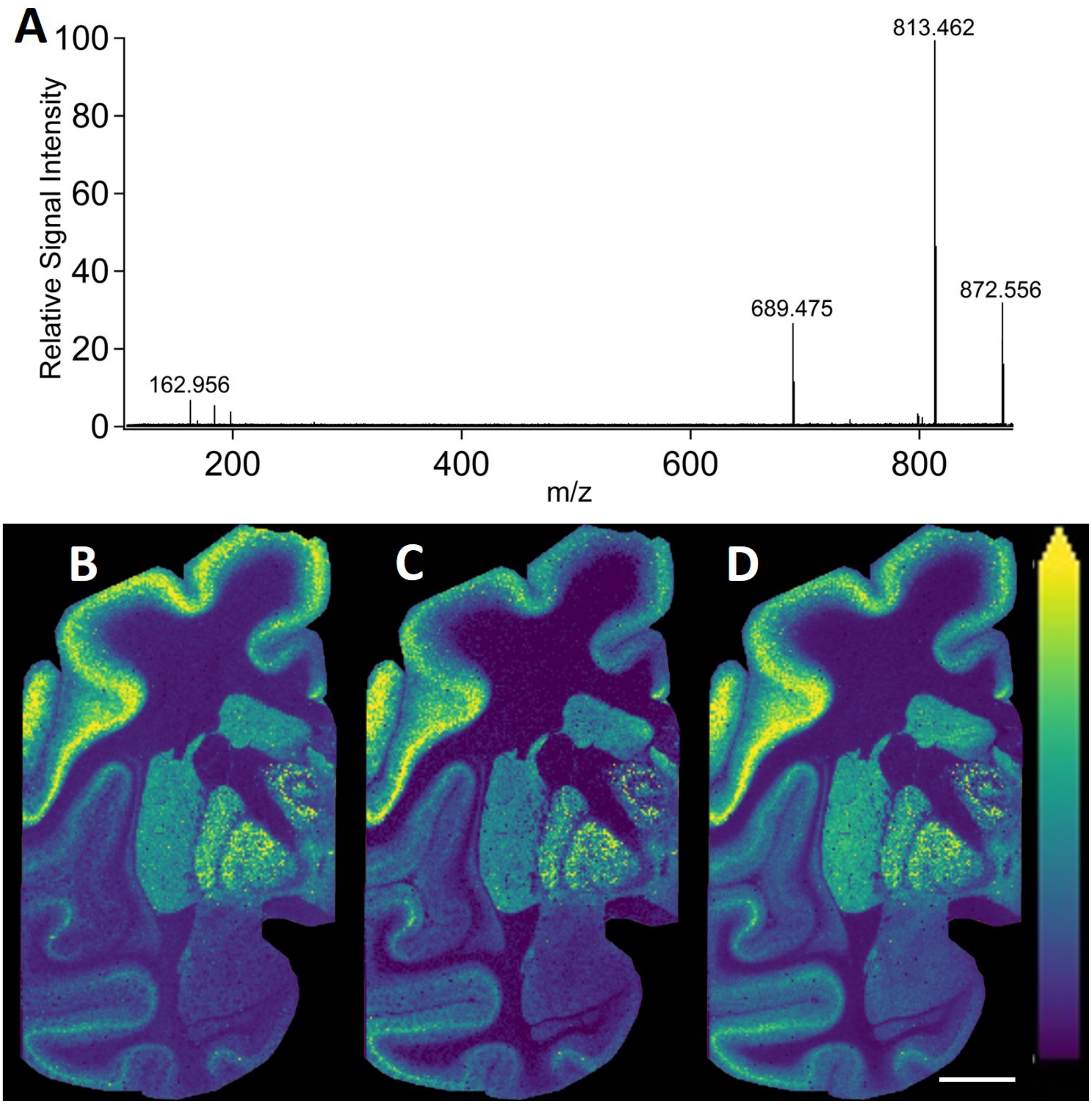

Supplementary Fig. 14. MALDI-MS/MS spectra obtained from a coronal macaque brain tissue section using MALDI-CID-FTICR. Precursor ion at **(a)** *m/z* 872.556. Product ions supporting the assignment of $[PC(40:6)+K]^+$ are at *m/z* 813.462 (loss of trimethylamine), *m/z* 689.475 (loss of phosphocholine head group) and *m/z* 162.956 (potassiated cyclophosphane). Due to the isolation width of 1 *m/z* unit, additional isobaric and isomeric lipids may have also fragmented, giving rise to additional product ions. MALDI-FTICR-MSI data showing similar ion distribution images of **(b)** $[PC(40:6)+H]^+$, **(c)** $[PC(40:6)+Na]^+$, and **(d)** $[PC(40:6)+K]^+$ in a control coronal macaque brain tissue section. All images are RMS-normalized and scaled to the maximum intensity of each ion. Lateral resolution: 150 µm; scale bar: 7 mm.

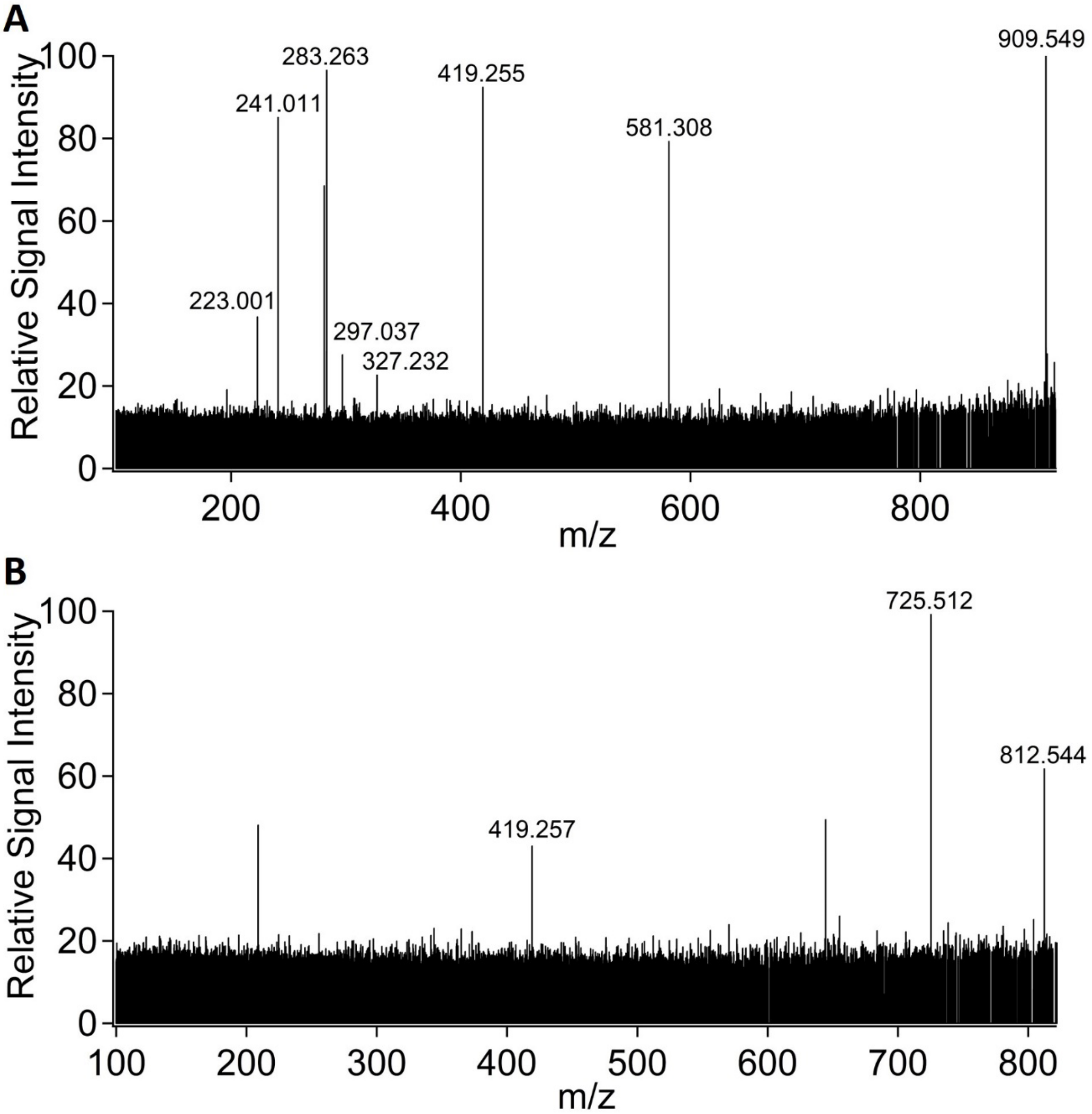

Supplementary Fig. 15. MALDI-MS/MS spectra obtained from macaque brain tissue sections using MALDI-CID-FTICR MS/MS. Precursor ions at **(a)** *m/z* 909.549, and **(b)** *m/z* 812.544. Product ions supporting the assignment of [PI (18:0/22:6)-H]$^-$ are at *m/z* 581.3008 (loss of sn2 acyl chain as ketene (RCH=C=O)), *m/z* 419.2523 (neutral loss of sn2 RCOOH group and inositol), *m/z* 297.0372 (glycerophosphoinositol-2H_2O), *m/z* 327.232 (sn2 RCOO$^-$ ion), *m/z* 283.263 (sn1 RCOO$^-$ ion), *m/z* 241.011 (inositol phosphate ion -H_2O) and *m/z* 223.0012 (inositol phosphate ion- 2H_2O). Product ions supporting the assignment of [PS(38:3)-H]$^-$ are at *m/z* 725.512 (loss of serine from precursor ion) and *m/z* 419.257 (neutral loss of sn2 RCOOH group and serine from [M-H]$^-$).

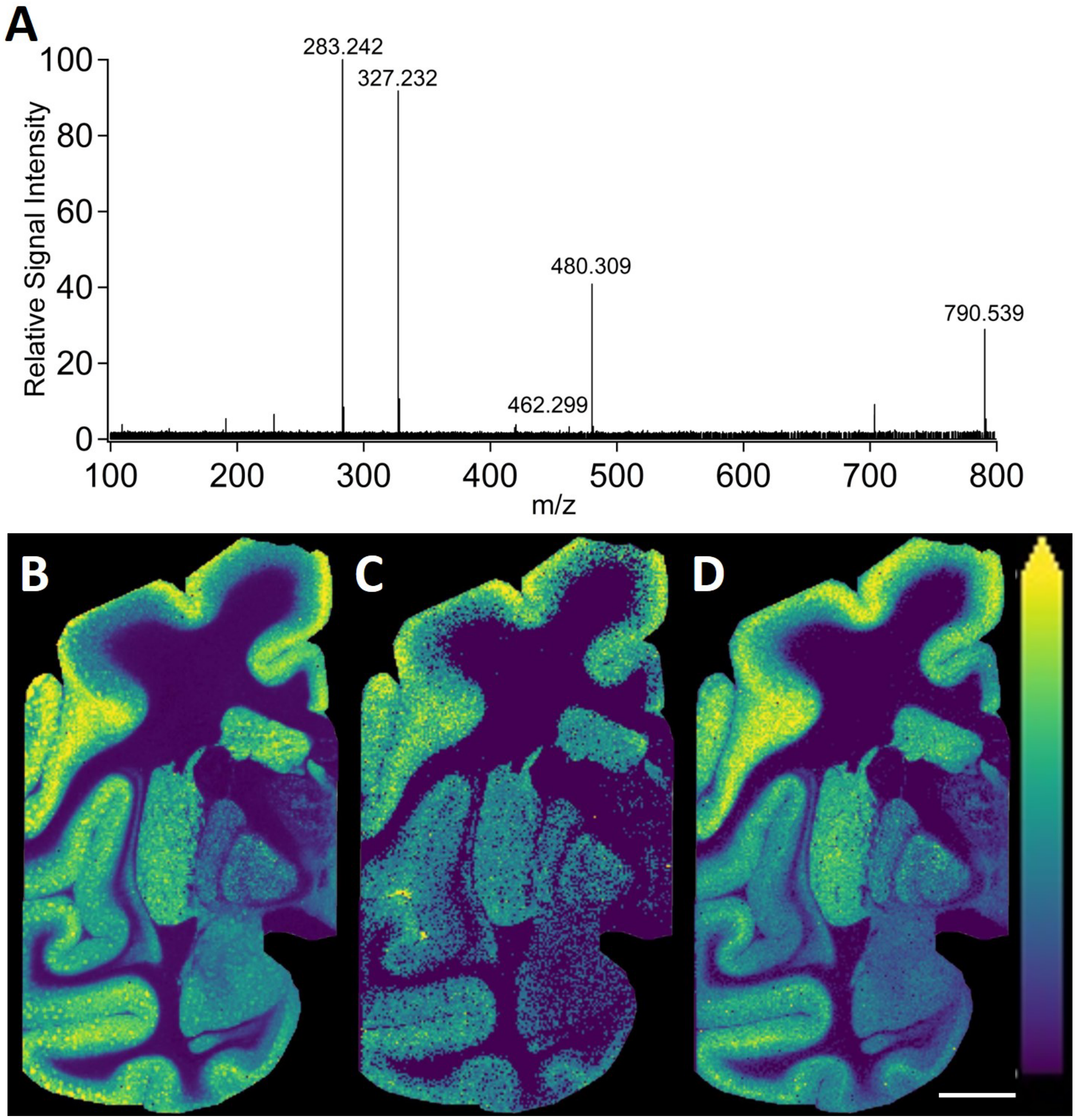

Supplementary Fig. 16. MALDI-MS/MS spectra obtained from a coronal macaque brain tissue section using MALDI-CID-FTICR. Precursor ion at **(a)** *m/z* 790.539. Product ions supporting the assignment of [PE (18:0/22:6)-H]$^-$ are at *m/z* 480.309 (loss of *sn*2 acyl chain as ketene, RCH=C=O), *m/z* 462.299 (neutral loss of *sn*2 RCOOH group), *m/z* 327.232 (*sn*2 RCOO$^-$ ion) and *m/z* 283.242 (*sn*1 RCOO$^-$ ion). Due to the isolation width of 1 *m/z* unit, additional isobaric and isomeric lipids may have also fragmented, giving rise to additional product ions. MALDI-FTICR-MSI data showing similar ion distribution images of **(b)** [PE (40:6)-H]$^-$, C) [PE (40:6)+Na]$^+$, and D) [PE (18:0/22:6)+K]$^+$ in a control coronal macaque brain tissue section. All images are RMS-normalized and scaled to the maximum intensity of each ion. Lateral resolution: 150 µm; scale bar: 7 mm.

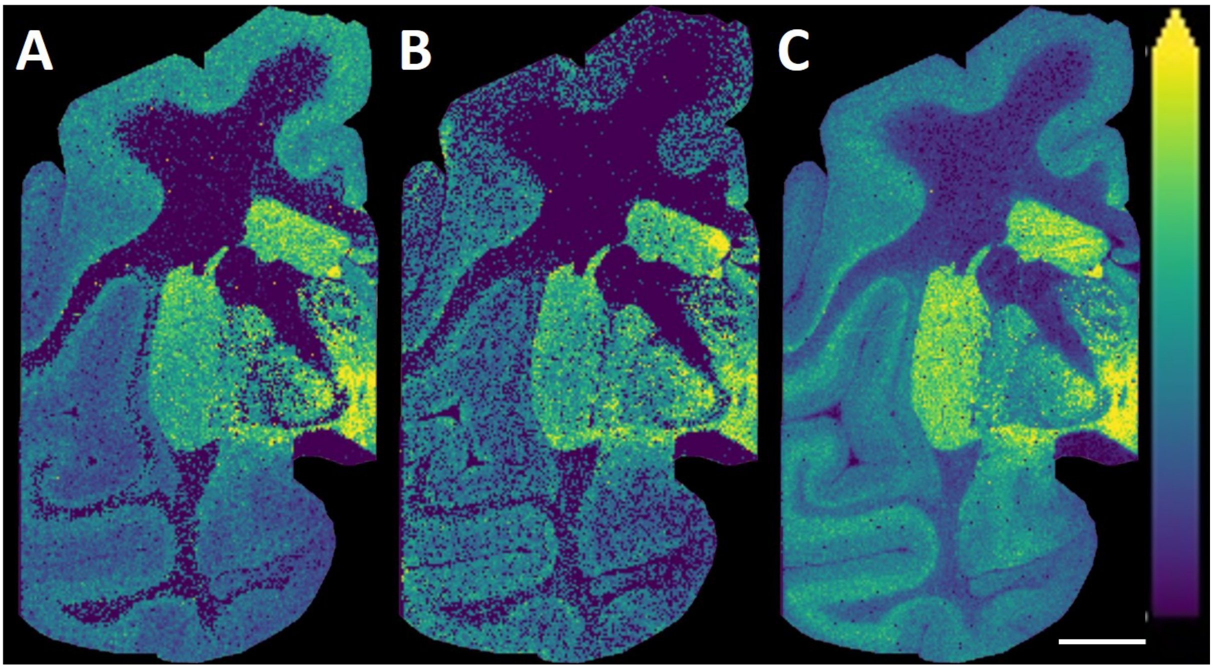

Supplementary Fig. 17. MALDI-FTICR-MSI data showing similar distributions of PC ion types. Ion images show the distributions of **(a)** $[PC(40:4)+H]^+$, **(b)** $[PC(40:4)+Na]^+$, and **(c)** $[PC(40:4)+K]^+$ in a control coronal macaque brain tissue section. All images are RMS-normalized and scaled to the maximum intensity of each ion. Lateral resolution: 150 μm; scale bar: 7 mm.

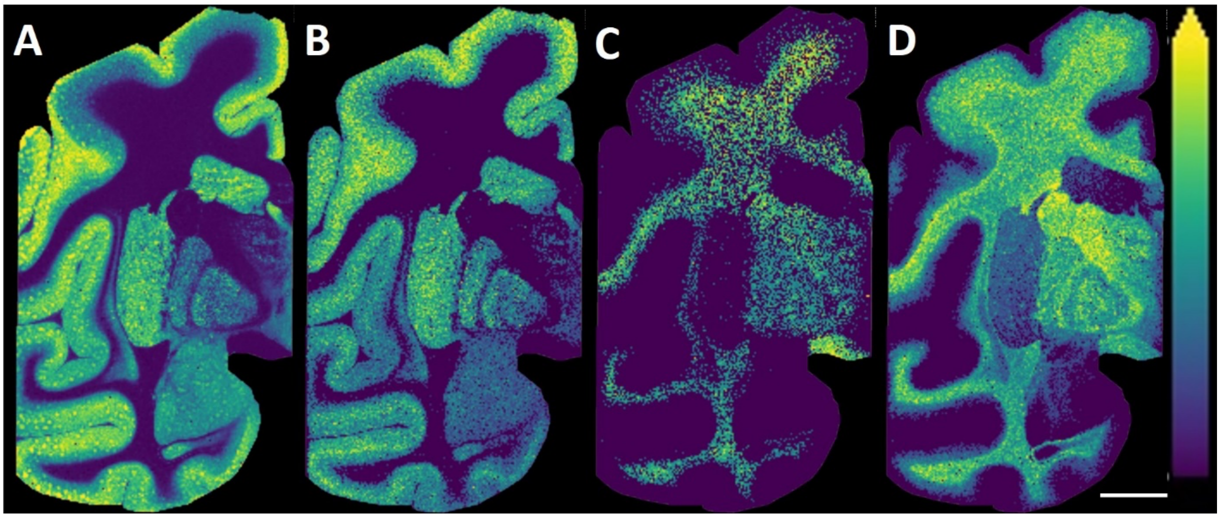

Supplementary Fig. 18. MALDI-FTICR-MSI data showing similar distributions of PE and HexCer ion types. Ion images show the distributions of **(a)** $[PE(P\text{-}18:0/22:6)\text{-}H]^-$, **(b)** $[PE\text{-}P(40:6)+K]^+$, and **(c)** $[HexCer(t42:2)+Na]^+$, **(d)** $[HexCer(t42:2)+K]^+$ in a control coronal macaque brain tissue section. All images are RMS-normalized and scaled to the maximum intensity of each ion. Lateral resolution: 150 μm; scale bar: 7 mm.

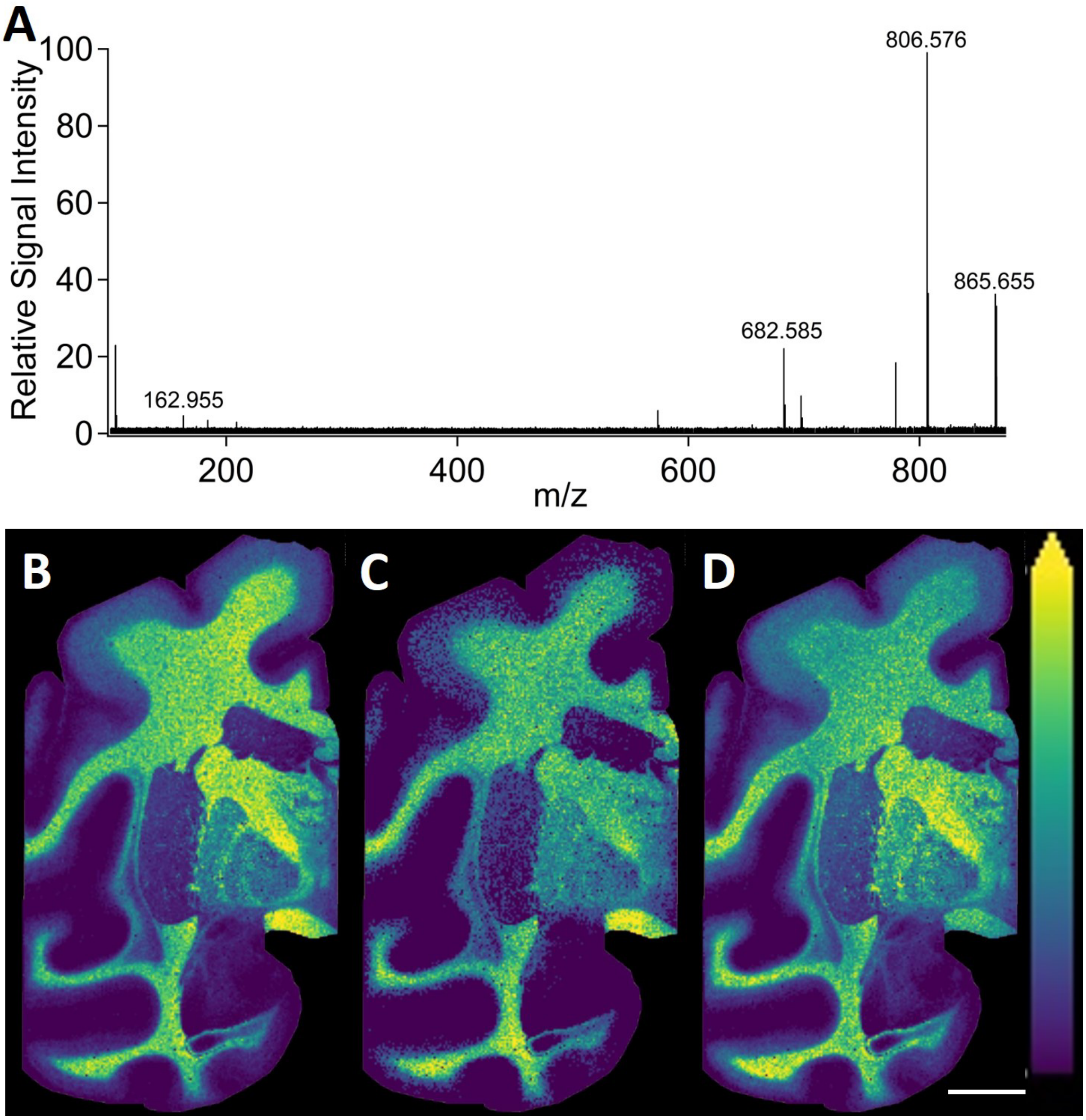

Supplementary Fig. 19. MALDI-MS/MS spectra obtained from coronal macaque brain tissue sections using MALDI-CID-FTICR. Precursor ions at **(a)** *m/z* 865.655. Product ions supporting the assignment of [SM(d43:2)+K]$^+$ are at *m/z* 806.576 (loss of trimethylamine), *m/z* 682.582 (loss of phosphocholine head group) and *m/z* 162.955 (potassiated cyclophosphane). Due to the isolation width of 1 *m/z* unit, additional isobaric and isomeric lipids may have also fragmented, giving rise to additional product ions. MALDI-FTICR-MSI data showing similar ion distributions images of **(b)** [SM(d43:2)+H]$^+$, **(c)** [SM(d43:2)+Na]$^+$, and **(d)** [SM(d43:2)+K]$^+$ in a control coronal macaque brain tissue section. All images are RMS-normalized and scaled to the maximum intensity of each ion. Lateral resolution: 150 µm; scale bar: 7 mm.

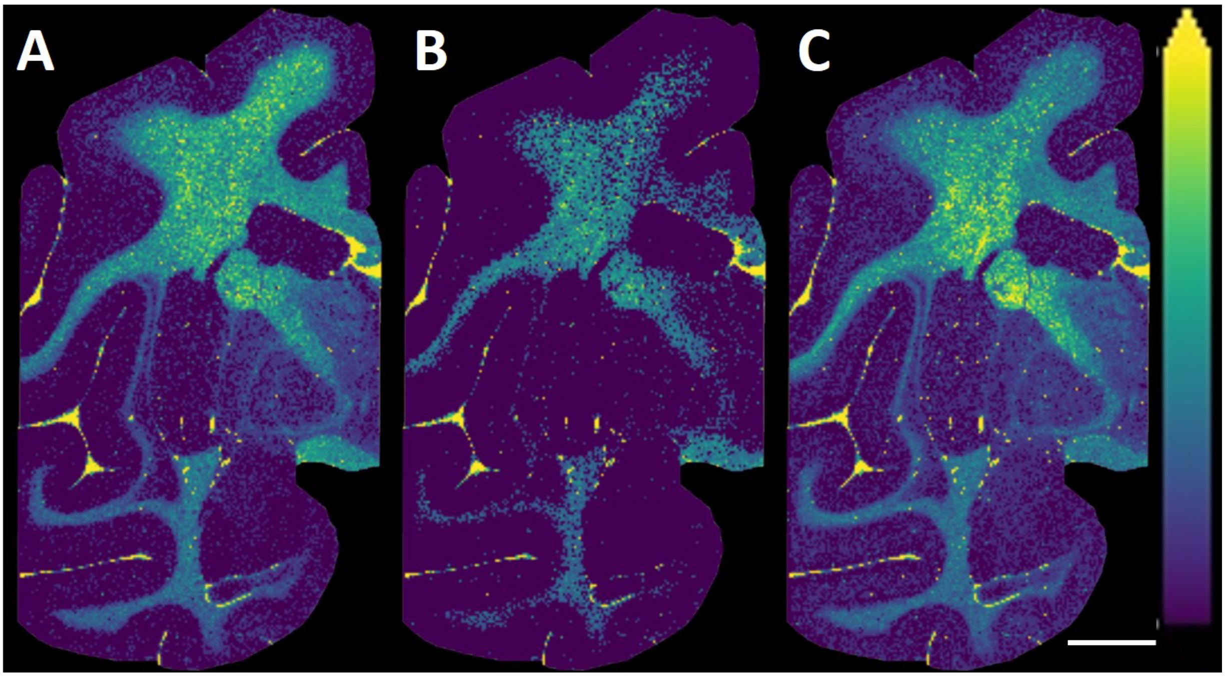

Supplementary Fig. 20. MALDI-FTICR-MSI data revealing similar distributions of SM ion types. Ion images show the distributions of **(a)** $[SM(d40{:}1)+H]^+$, **(b)** $[SM(d40{:}1)+Na]^+$, and **(c)** $[SM(d40{:}1)+K]^+$ in a control coronal macaque brain tissue section. All images are RMS-normalized and scaled to the maximum intensity of each ion. Lateral resolution: 150 µm; scale bar: 7 mm.

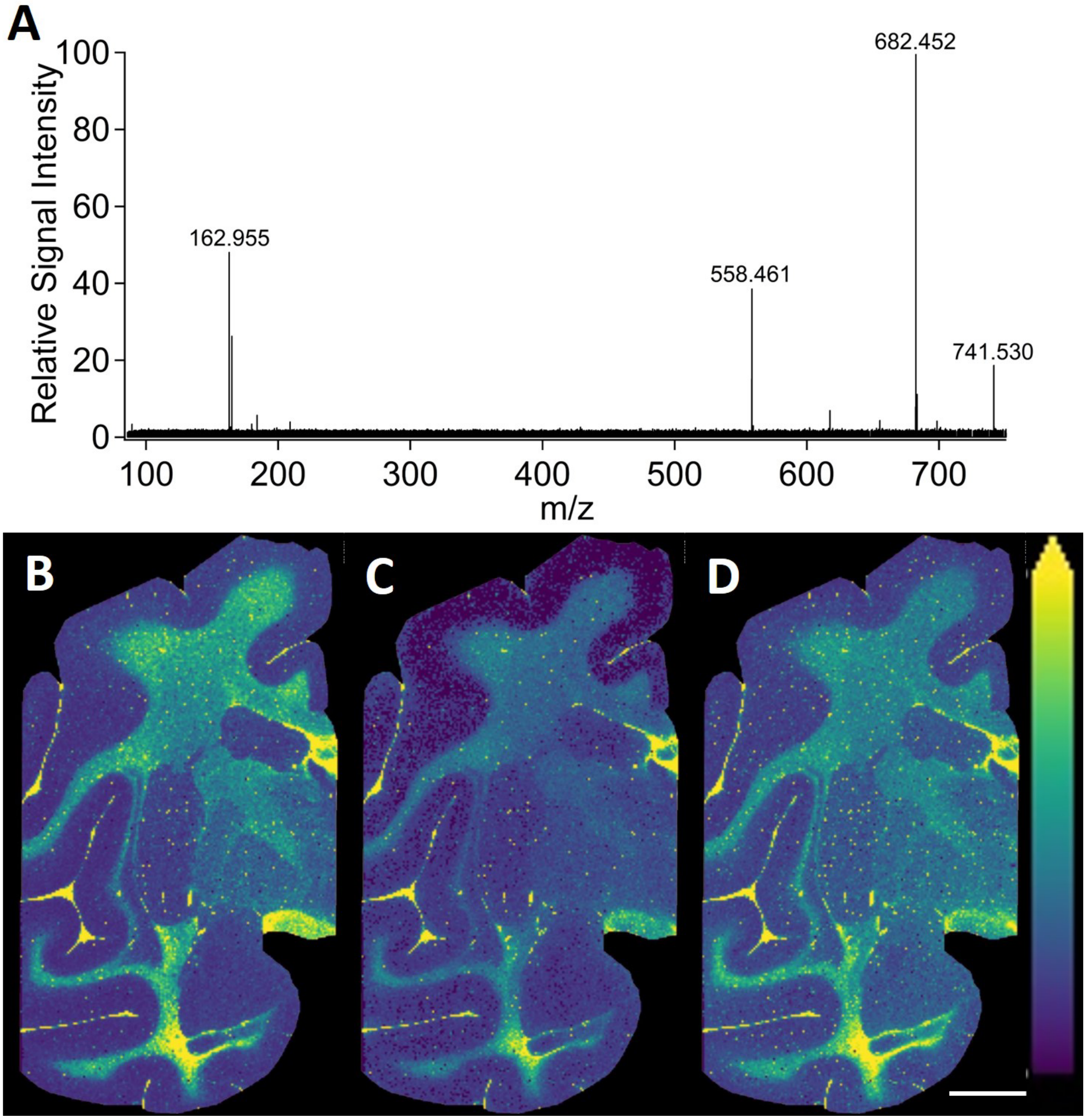

Supplementary Fig. 21. MALDI-MS/MS spectra obtained from coronal macaque brain tissue sections using MALDI-CID-FTICR. Precursor ions at **(a)** *m/z* 741.530. Product ions supporting the assignment of $[SM(d34:1)+K]^+$ are at *m/z* 682.452 (loss of trimethylamine), *m/z* 558.461 (loss of phosphocholine head group) and *m/z* 162.955 (potassiated cyclophosphane). Due to the isolation width of 1 *m/z* unit, additional isobaric and isomeric lipids may have also fragmented, giving rise to additional product ions. MALDI-FTICR-MSI data revealing similar ion distributions images of **(b)** $[SM(d34:1)+H]^+$, **(c)** $[SM(d34:1)+Na]^+$, and **(d)** $[SM(d34:1)+K]^+$ in a control coronal macaque brain tissue section. All images are RMS-normalized and scaled to the maximum intensity of each ion. Lateral resolution: 150 µm; scale bar: 7 mm.

Supplementary Table 1. List of annotated lipid species showing significant differences in multiple two-tailed *t*-tests between non-LID vs. LID and Ctrl vs. MPTP groups in multiple brain regions.

Several *m/z* values were found to contribute ($p \leq 0.05$) in volcano plots to the separation of the compared groups and were selected for further identification. Abbreviations: PoG: postcentral gyrus; PrG: precentral gyrus; ACgG: anterior cingulate gyrus; Cd: caudate nucleus; Ins: insula; Cl: claustrum; Put: putamen; GPe/GPi: globus pallidus externa/interna; tw: temporal white matter.

**GPi**			
***p* value**	**logFC_LID-nonLID**	***m/z***	**Annotations**
0,003076797	0,175815353	850.56125	PC(38:4)+K-3rd isotope
0,007278913	-0,116057082	850.57053	PC(38:3)+K
0,008159641	-0,097239803	747.6097	PC-P(34:1)-2nd isotope
0,010746091	-0,103995575	851.57638	PC(38:3)+K-2nd isotope
0,012128161	-0,092751094	746.6079	PC-P(34:1)
0,013893836	-0,276612931	748.61299	PC-P(34:1)-3rd isotope
0,017064416	-0,151700354	774.63849	PC-P(36:1)
0,020652387	0,085842083	761.45381	PA(38:5)+K
0,022583243	-0,282922572	775.6411	PC-P(36:1)-2nd isotope
0,025623091	-0,08823994	822.54118	PC(36:3)+K
0,026723896	0,075765426	805.55395	PC(36:4)+Na-2nd isotope
0,027606554	-0,093676113	768.58887	PC-P(34:1)+Na
0,028250729	0,156833316	822.52334	PC(36:4)+K-3rd isotope
0,031440524	0,075770663	857.58681	PC(40:6)+Na-2nd isotope
0,035233516	0,086107086	821.52769	PC(36:4)+K-2nd isotope
0,036439585	0,080458471	820.52726	PC(36:4)+K
0,036958324	0,287107229	762.45611	PA(38:5)+K-2nd isotope
0,037325956	0,121933308	848.61829	PC(38:4)+K
0,040389855	-0,04084938	784.52481	Unknown
0,04058579	0,31384633	851.55829	PC(38:4)+K-4th isotope
0,040601436	-0,041261421	785.53077	Unkown
0,042085684	0,071356556	856.58503	PC(40:6)+Na
0,042303901	-0,095115346	784.5634	PC-P(34:1)+K
0,04379585	0,303549349	823.5301	PC(36:4)+K-3rd isotope
0,044768657	0,082210067	804.55328	PC(36:4)+Na
0,003351847	0,142957278	909.55327	PI (40:6)
0,004102122	0,142375627	910.55453	PI (40:6)-2nd isotope
0,00593701	-0,1421167	725.5124	PA(38:3)
0,008385786	0,251914335	911.5541	PI (40:6)-3rd isotope
0,009925543	0,267817912	907.53414	PI (40:7)
0,024686224	-0,224805672	768.5548	PE(38:3)
0,030043511	-0,121210768	724.53016	PE-O-36:4
0,041071329	-0,168548671	812.54542	PS(38:3)
0,043782442	-0,167031935	813.54788	PS(38:3)-2nd isotope
0,045246097	0,344824744	912.56954	PI(40:6)-4th isotope
0,046274109	-0,061757921	876.59059	SHexCer(t40:2)
0,047752296	-0,311846946	726.51797	PA(38:3)-2nd isotope
0,049923658	-0,074901589	877.59407	SHexCer(t40:2)-2nd isotope
**GPe**			
***p* value**	**logFC_LID-nonLID**	***m/z***	**Annotations**
0,020599929	0,092566946	825.62485	SM(d40:1)+K
0,035602196	0,15382485	826.62949	SM(d40:1)+K-2nd isotope
0,036790222	-0,194239	768.5548	PE(38:3)
0,039595069	-0,078659831	822.54118	PC(36:3)+K
0,041706811	-0,095866575	850.57053	PC(38:3)+K

0,045951589	0,178258829	907.53414	PI(40:7)
0,046792365	-0,104539808	725.5124	PA(38:3)
**Put**			
***p* value**	**logFC_LID-nonLID**	***m/z***	**Annotation**
0,009678364	-0,129386055	843.659	unkown
0,017494053	-0,144311017	850.57053	PC(38:3)+K
0,03317444	-0,071572641	822.54118	PC(36:3)+K
0,04397157	-0,237106074	844.66431	unkown
**Cl**			
***p* value**	**logFC_LID-nonLID**	***m/z***	**Annotation**
0,006106585	-0,071029188	746.6079	PC-P(34:0)
0,007608806	-0,260246892	774.63849	PC-P(36:0)
0,008123685	-0,07317537	747.6097	PC-P(34:0)-2nd isotope
0,015860026	-0,291632923	773.62483	PC-P(36:1)-2nd isotope
0,016527832	-0,162763224	772.62274	PC-P(36:1)
0,020573163	-0,064633754	810.58086	PC-P(36:1)+K
**Ins**			
***p* value**	**logFC_LID-nonLID**	***m/z***	**Annotation**
0,012801783	-0,077900746	741.53194	SM(d34:1)
0,015765671	-0,068959507	742.53508	SM(d34:1)-2nd isotope
0,021121153	-0,060764227	822.54118	PC(36:3)+K
0,026557393	-0,288551839	743.53819	SM(d34:1)-3rd isotope
0,034239622	-0,057301375	823.54702	PC(36:3)+K-2nd isotope
**Cd**			
***p* value**	**logFC_LID-nonLID**	***m/z***	**Annotation**
0,010464252	-0,0771097	822.54118	PC(36:3)+K
0,012503707	-0,111162964	843.659	Uknown
0,017250628	-0,132781487	850.57053	PC(38:3)+K
0,034888679	-0,095649546	725.5124	PA(38:3)
0,035335976	-0,078006218	851.57638	PC(38:3)+K-2nd isotope
0,036171892	0,045188717	860.46981	Unkown
0,040943373	-0,067077877	823.54702	PC(36:3)+K-2nd isotope
**ACgG**			
***p* value**	**logFC_LID-nonLID**	***m/z***	**Annotation**
0,007565143	0,074053915	836.5404	Unknown
0,022250056	-0,056678524	822.54118	PC(36:3)+K
0,025989646	0,366523374	837.55797	Unknown
0,041377326	0,156707016	858.52926	Unknown
0,045857633	-0,079428722	850.57053	PC(38:3)+K
**tw**			
***p* value**	**logFC_LID-nonLID**	***m/z***	**Annotation**
0,047408695	-0,085301061	850.57053	PC(38:3)+K
0,047464042	-0,074079312	851.57638	PC(38:3)+K-2nd isotope
**PrG**			
***p* value**	**logFC_LID-nonLID**	***m/z***	**Annotation**
0,009158103	0,08638251	860.46981	Unknown
0,027038527	-0,122861436	725.5124	PA(38:3)
0,030111349	-0,04058053	785.53077	Unknown
0,030375442	-0,053499354	747.6097	PC-P(34:0)-2nd isotope
0,031017885	-0,352714826	774.63849	PC-P(36:0)
0,035004594	-0,038585928	784.52481	Unknown
0,036756729	-0,053438588	746.6079	PC-P(34:0)
0,039630367	-0,063378495	822.54118	PC(36:3)+K
0,040419411	0,157294788	739.51293	Unknown

0,048601961	-0,082928946	850.57053	PC(38:3)+K
**PoG**			
***p* value**	**logFC_LID-nonLID**	***m/z***	**Annotation**
0,017670759	0,119177709	863.61274	Unknown
0,021612872	0,081338648	860.59498	SHexCer(d40:2)
0,022034941	-0,067217395	822.54118	PC(36:3)+K
0,023736742	-0,294858771	773.62483	Unknown
0,024593819	0,101697565	862.60719	SHexCer(d40:1)
0,025874099	0,09796937	861.59642	SHexCer(d40:2)-2nd isotope
0,026098129	-0,216649726	774.63849	PC-P(36:0)
0,026204651	-0,272723125	831.69499	Unknown
0,028513397	-0,113058849	810.54341	unknown
0,029741861	-0,419904913	775.6411	PC-P(36:0)-2nd isotope
0,036562236	-0,248913551	876.51904	Unknown
**GPi**			
***p* value**	**logFC_MPTP-CTRL**	***m/z***	**Annotation**
0,023471135	0,153266417	862.60719	SHexCer(d40:1)
0,025888019	0,164301496	863.61274	SHexCer(d40:1)-2nd isotope
0,034525447	-0,153839863	919.64157	SHexCer(t43:2)-2nd isotope
0,036903012	-0,144638205	918.63675	SHexCer(t43:2)
0,042128159	-0,175421927	802.57436	unknown
0,047290939	0,108944375	860.59498	SHexCer(d40:2)
0,000857655	-0,113097099	864.63511	HexCer(t42:2)+K
0,003173458	-0,206863953	865.63607	HexCer(t42:2)+K-2ndisotope
0,005171351	-0,307677067	848.66088	HexCer(t42:2)+Na
0,013500187	-0,059312167	853.59262	PC(38:2)+K-2nd isotope
0,016704191	-0,074700287	830.51187	PE(40:6)+K
0,023463416	-0,087941817	802.56166	Unknown
0,025073731	-0,087174621	801.5597	Unknown
0,02839916	-0,086747002	831.51415	PE(40:6)+K-2nd isotope
0,028400752	-0,150533265	742.48755	Unknown
0,028802429	-0,164295313	803.55969	Unknown
0,031532227	-0,057163484	852.59082	PC(38:2)+K
0,038041944	0,34534366	868.66474	Unknown
0,039368009	0,277288944	867.66052	Unknown
0,047166094	-0,095958942	865.65689	SM(d43:2)+K
**GPe**			
***p* value**	**logFC_MPTP-CTRL**	***m/z***	**Annotation**
0,004723858	-0,095610051	864.63511	HexCer(t42:2)+K
0,009166978	-0,191779013	898.57327	PC(42:7)+K
0,009367307	-0,076358991	810.54341	Unknown
0,009915604	-0,232593013	976.64589	Uknown
0,013433958	-0,066805786	830.51187	PE(40:6)+K
0,01579976	-0,162308112	865.63607	HexCer(t42:2)+K-2ndisotope
0,016017513	-0,071491169	831.51415	PE(40:6)+K -2nd isotope
0,018278925	-0,081025732	854.5666	PC(40:7)+Na
0,021987303	-0,105677167	866.65862	SM(d43:2)+K-2nd isotope
0,022465977	-0,243409091	848.66088	HexCer(t42:2)+Na
0,022616555	0,140352623	862.60719	SHexCer(d40:1)
0,023766844	-0,113458397	865.65689	SM(d43:2)+K
0,02436898	0,146984193	863.61274	SHexCer(d40:1)-2nd isotope
0,03075964	-0,135436868	805.5932	PE-O(42:6)-2nd isotope
0,03181941	-0,244691937	850.68395	SM(d43:2)+Na-2nd isotope
0,032180529	-0,137001455	804.59251	PE-O(42:6)
0,034367052	-0,118887673	849.68279	SM(d43:2)+Na
0,034623338	-0,059283344	853.59262	PC(38:2)+K-2nd isotope

0,035756089	-0,126492633	1004.67833	Unknown
0,043655063	-0,094173743	860.61631	PC(40:4)+Na
0,045546798	-0,055910861	852.59082	PC(38:2)+K
0,046155056	-0,117417718	814.5154	PE-P(40:6)+K
0,04640152	-0,286528067	916.52433	PC(44:12)+K

Supplementary Table 2. List of assigned lipid species with high mass accuracy obtained from MALDI-FTICR-MSI experiments.

A search of *m/z* values with 0.01 *m/z* mass tolerance in LIPID MAPS was conducted for both negative and positive polarities, including all the ion types. Lipid species marked with two asterisks (**) were identified using mass accuracy, MS/MS and/or by comparing the observed distributions of different adducts of the same molecule across the coronal macaque brain tissue sections. Lipids marked with one asterisk (*) were identified based solely on mass accuracy since the peak intensity was too low for MS/MS analysis of the tissue. For ion species for which specific information about the fatty acid chains of the lipids was obtained, this information is included in the annotations. For species identified solely based on head group fragments or mass accuracy, a combined isomer annotation is provided. PC plasmalogens were initially assigned as PC species based on the head group information from MS/MS analysis and by comparing the observed distributions of different adducts of the same molecule across the coronal macaque brain tissue sections. Further differentiation between plasmanyl and plasmenyl residues in the ether lipid structure was made based on literature showing that in mammals, the ether bond of plasmalogens is located at the *sn*-1 position of the glycerol backbone,[1-4] typically substituted with a saturated or mono-unsaturated alkyl/alkenyl residue.[3] Identified lipids were grouped into four main categories: i) plasmalogen phosphatidylcholines (PCs), ii) polyunsaturated fatty acid (PUFA)-containing glycerophospholipids (GPLs), iii) hydroxylated (t) sphingolipids, and iv) non-hydroxylated (d) sphingolipids. The h sign in the fatty acid substituent of sulfatides among sphingolipids indicates their hydroxylation. Lipid species assignments presented in italic text support the identification of other lipid species in the table.

Lipid Species Assignment	Ion Type	Formula	*m/z* Theoretical	*m/z* Observed	ppm Error
**Plasmalogen PCs**					
PC-P(34:0)**	$[M+H]^+$	C42H84NO7P	746,605817	746,60628	0,620
PC-P(34:0)**	$[M+Na]^+$	C42H84NO7PNa	768,587762	768,58817	0,531
PC-P(34:0)**	$[M+K]^+$	C42H84NO7PK	784,561699	784,56119	-0,649
PC-P(36:0)**	$[M+H]^+$	C44H88NO7P	774,637118	774,63833	1,565
PC-P(36:0)**	$[M+K]^+$	C44H88NO7PK	812,592999	812,59336	0,444
PC-P(36:1)**	$[M+H]^+$	C44H86NO7P	772,621468	772,62261	1,478
PC-P(36:1)**	$[M+K]^+$	C44H86NO7PK	810,577349	810,57828	1,149
**PUFA-containing GPLs**					
PC(38:2)*	$[M+K]^+$	C46H88NO8PK	852,587914	852,58882	1,063
PC(38:3)**	$[M+K]^+$	C46H86NO8PK	850,572264	850,57111	-1,357
*PC(38:3)***	*$[M+H]^+$*	*C46H87NO8P*	*813,624207*	*813,62222*	*-2,442*
PA(38:5)+K*	$[M+K]^+$	C41H71O8PK	761,451814	761,4531	1,689
PC(36:3)*	$[M+K]^+$	C44H82NO8PK	822,540964	822,54102	0,068
PC(36:4)**	$[M+K]^+$	C44H80NO8PK	820,525313	820,52595	0,776
PC(36:4)**	$[M+Na]^+$	C44H80NO8PNa	804,551376	804,55267	1,608
PC(38:4)**	$[M+K]^+$	C46H84NO8PK	848,556614	848,55793	1,551
PC(38:4)**	$[M+Na]^+$	C46H84NO8PNa	832,582676	832,58252	-0,187
PC(40:6)**	$[M+Na]^+$	C48H84NO8PNa	856,582676	856,58383	1,347
*PC(40:6)***	*$[M+K]^+$*	*C48H84NO8PK*	*872,556614*	*872,55752*	*1,038*
*PC(40:6)***	*[M+H]+*	*C48H84NO8P*	*834,600732*	*834,60122*	*0,585*
PC(40:7)**	$[M+Na]^+$	C48H82NO8PNa	854,567026	854,56639	-0,744
*PC(40:7)***	*$[M+K]^+$*	*C48H82NO8PK*	*870,540964*	*870,5401*	*-0,992*
PI(18:0/22:6)**	$[M-H]^-$	C49H83O13P	909,549853	909,55171	2,042

PI(40:7)*	[M-H]$^-$	C49H81O13P	907,534203	907,53426	0,063
PE(40:6)**	[M+K]$^+$	C45H78NO8PK	830,509663	830,51092	1,514
PE(40:6)**	[M+Na]$^+$	C45H78NO8PNa	814,535726	814,53578	0,066
PE(18:0/22:6)**	[M-H]$^-$	C45H78NO8P	790,539229	790,53819	-1,314
PC(40:4)**	[M+Na]$^+$	C48H88NO8PNa	860,613976	860,61555	1,829
PC(40:4)**	[M+K]$^+$	C48H88NO8PK	876,587914	876,58919	1,456
PC(40:4)**	[M+H]$^+$	C48H88NO8P	838,632032	838,63135	-0,813
PE-P(40:6)**	[M+K]$^+$	C45H78NO7PK	814,514749	814,51476	0,014
PE(P-18:0/22:6)**	[M-H]$^-$	C45H78NO7P	774,544314	774,54509	1,002
PE-O(42:6)*	[M-H]$^-$	C47H84NO7P	804,591265	804,59169	0,528
PC(42:7)*	[M+K]$^+$	C50H86NO8PK	898,572264	898,57297	0,786
PC(44:12)*	[M+K]$^+$	C52H80NO8PK	916,525313	916,52413	-1,291
PA(38:3)*	[M-H]$^-$	C41H75O8P	725,51268	725,51223	-0,620
PE-O(36:4)*	[M-H]$^-$	C41H76NO7P	724,528664	724,5298	1,568
PS(38:3)**	[M-H]$^-$	C44H80NO10P	812,544708	812,54422	-0,601
PE(38:3)**	[M-H]$^-$	C43H80NO8P	768,554879	768,55537	0,639
**Sphingolipids**					
SHexCer(d18:1/h22:1)**	[M-H]$^-$	C46H87NO12S	876,587622	876,58925	1,857
SHexCer(d18:1/h25:1)**	[M-H]$^-$	C49H93NO12S	918,634572	918,63674	2,360
HexCer(t42:2)**	[M+K]$^+$	C48H91NO9K	864,632541	864,63357	1,190
HexCer(t42:2)**	[M+Na]$^+$	C48H91NO9Na	848,658604	848,65969	1,280
SHexCer(d40:1)**	[M-H]$^-$	C46H89NO11S	862,608357	862,60735	-1,167
SHexCer(d40:2)**	[M-H]$^-$	C46H87NO11S	860,592707	860,59401	1,514
SM(d43:2)**	[M+K]$^+$	C48H95N2O6PK	865,655934	865,65578	-0,178
SM(d43:2)**	[M+Na]$^+$	C48H95N2O6PNa	849,681996	849,68236	0,428
SM(d43:2)**	[M+H]$^+$	C48H95N2O6P	827,700052	827,7002	0,179
SM(d40:1)**	[M+K]$^+$	C45H91N2O6PK	825,624634	825,62416	-0,574
SM(d40:1)**	[M+Na]$^+$	C45H91N2O6PNa	809,650696	809,65097	0,338
SM(d40:1)**	[M+H]$^+$	C45H91N2O6P	787,668752	787,66813	-0,790
SM(d34:1)**	[M+K]$^+$	C39H79N2O6PK	741,530733	741,53188	1,547
SM(d34:1)**	[M+Na]$^+$	C39H79N2O6PNa	725,556796	725,55722	0,584
SM(d34:1)**	[M+H]$^+$	C39H79N2O6P	703,574852	703,57589	1,475

Supplementary Table 3. Detailed explanation of lipid species assignments.

List of all putative lipid species assigned to each entry in Supplementary Table 2 from the LIPID MAPS database with 0.01 *m/z* mass tolerance. Lipid species included in Supplementary Table 2 after structural validation are indicated in bold font. Specifically, lipid species presented only in bold were identified based on MS/MS and/or by comparing the observed distributions of different adducts of the same molecule across the coronal brain tissue sections. Lipid species assignments presented in italic bold were identified solely based on mass accuracy. SHexCer species were annotated based on our previous lipidomics study of hydroxylated and non-hydroxylated SHexCer species.[5]

Input Mass	Matched Mass	ppm Errors	Name	Formula	Ion	Comment
746.60628	746.6058	0.64	PE O-37:1	C42H84NO7P	[M+H]+	
**746.60628**	**746.6058**	**0.64**	**PC-P(34:0)**	**C42H84NO7P**	**[M+H]+**	**MS/MS (see SI Fig. 9)**
746.60628	746.6058	0.64	PA O-39:2	C42H81O7P	[M+NH4]+	
**768.58817**	**768.587762**	**0.53**	**PC-P(34:0)**	**C42H84NO7PNa**	**[M+Na]+**	**similar distribution with M+H and K adduct ion (see SI Fig. 9)**
768.58817	768.5902	-2.64	PC O-36:4	C44H82NO7P	[M+H]+	
768.58817	768.5902	-2.64	PC 36:2	C44H84NO8P	[M+H-H2O]+	
768.58817	768.5902	-2.64	PE 39:2	C44H84NO8P	[M+H-H2O]+	
**784.56119**	**784.561699**	**-0.65**	**PC-P(34:0)**	**C42H84NO7PK**	**[M+K]+**	**similar distribution with M+H and Na adduct ion (see SI Fig. 9)**
784.56119	784.5569	5.47	GlcADG 34:3	C43H74O11	[M+NH4]+	
784.56119	784.5569	5.47	MGDG 34:4;O	C43H74O11	[M+NH4]+	
784.56119	784.5674	-7.92	HexCer 37:2;O2	C43H81NO8Na2	[M+2Na-H]+	
**774.63833**	**774.6371**	**1.59**	**PC-P(36:0)**	**C44H88NO7P**	**[M+H]+**	**similar distribution with K adduct ion (see SI Fig. 10)**
774.63833	774.6371	1.59	PE O-39:1	C44H88NO7P	[M+H]+	
774.63833	774.6454	-9.13	HexCer 38:0;O3	C44H87NO9	[M+H]+	
**812.59336**	**812.592999**	**0.44**	**PC-P(36:0)**	**C42H84NO7PK**	**[M+K]+**	**similar distribution with M+H ion (see SI Fig. 10)**
812.59336	812.5904	3.64	PC dO-37:4	C45H86NO6PNa2	[M+2Na-H]+	
812.59336	812.5987	-6.57	HexCer 39:2;O2	C45H85NO8Na2	[M+2Na-H]+	
**772.62261**	**772.6215**	**1.44**	**PC-P(36:1)**	**C44H86NO7P**	**[M+H]+**	**similar distribution with K adduct**

						**ion (see SI Fig. 10)**
772.62261	772.6215	1.44	PE O-39:2	C44H86NO7P	[M+H]+	
772.62261	772.6297	-9.18	HexCer 38:1;O3	C44H85NO9	[M+H]+	
772.62261	772.6215	1.44	PC 36:0	C44H88NO8P	[M+H-H2O]+	
772.62261	772.6215	1.44	PE 39:0	C44H88NO8P	[M+H-H2O]+	
772.62261	772.6297	-9.18	HexCer 38:0;O4	C44H87NO10	[M+H-H2O]+	
**810.57828**	**810.577349**	**1.15**	**PC-P(36:1)**	**C44H86NO7PK**	**[M+K]+**	**similar distribution with M+H ion (see SI Fig. 10)**
810.57828	810.5855	-8.91	IPC 36:0;O2	C42H84NO11P	[M+H]+	
852.58882	852.5865	2.72	SHexCer 38:1;O3	C44H85NO12S	[M+H]+	
852.58882	852.596	-8.42	IPC 38:0;O4	C44H88NO13P	[M+H-H2O]+	
852.58882	852.596	-8.42	PI O-35:2	C44H83O12P	[M+NH4]+	
852.58882	***852.587914***	***1.06***	***PC 38:2***	***C46H88NO8PK***	***[M+K]+***	***mass accuracy***
852.58882	852.5879	1.08	PE 41:2	C46H88NO8PK	[M+K]+	
852.58882	852.5854	4.01	PE O-42:5	C47H86NO7PNa2	[M+2Na-H]+	
850.57111	850.5804	-10.92	PI O-35:3	C44H81O12P	[M+NH4]+	
**850.57111**	**850.572264**	**-1.36**	**PC 38:3**	**C46H86NO8PK**	**[M+K]+**	**similar distribution with M+H ion (see SI Fig. 11)**
850.57111	850.5723	-1.40	PE 41:3	C46H86NO8PK	[M+K]+	
850.57111	850.5697	1.66	PE O-42:6	C47H84NO7PNa2	[M+2Na-H]+	
**813.62222**	**813.624207**	**-2.44**	**PC 38:3**	**C46H87NO8P**	**[M+H]+**	**similar distribution with K adduct ion (see SI Fig. 11)**
813.62222	813.6246	-2.93	SM 39:0;O2	C44H91N2O6PK	[M+K]+	
813.62222	813.6134	10.84	PA O-42:0	C45H91O7PK	[M+K]+	
761.4531	761.4599	-8.93	PI 30:2	C39H71O13P	[M+H-H2O]+	
761.4531	761.4575	-5.78	PI O-28:1	C37H71O12PNa	[M+Na]+	
761.4531	761.448	6.70	SQDG 28:0	C37H70O12SNa	[M+Na]+	
761.4531	***761.451814***	***1.69***	***PA (38:5)***	***C41H71O8PK***	***[M+K]+***	***mass accuracy***
822.54102	822.5432	-2.65	PE 44:10	C49H78NO8P	[M+H-H2O]+	
822.54102	822.5491	-9.82	PI O-33:3	C42H77O12P	[M+NH4]+	
822.54102	***822.540964***	***0.07***	***PC 36:3***	***C44H82NO8PK***	***[M+K]+***	***mass accuracy***
822.54102	822.541	0.02	PE 39:3	C44H82NO8PK	[M+K]+	
822.54102	822.5384	3.19	PE O-40:6	C45H80NO7PNa2	[M+2Na-H]+	
820.52595	820.531	-6.15	IPC 34:0;O3	C40H80NO12PNa	[M+Na]+	
820.52595	820.5205	6.64	MGDG 36:8;O2	C45H70O12	[M+NH4]+	

**820.52595**	**820.525313**	**0.78**	**PC 36:4**	**C44H80NO8PK**	**[M+K]+**	**MS/MS (see Fig. 11-12)**
820.52595	820.5253	0.79	PE 39:4	C44H80NO8PK	[M+K]+	
820.52595	820.5228	3.84	PE O-40:7	C45H78NO7PNa2	[M+2Na-H]+	
**804.55267**	**804.551376**	**1.61**	**PC 36:4**	**C44H80NO8PNa**	**[M+Na]+**	**similar distribution with K adduct ion (see SI Fig. 11)**
804.55267	804.5538	-1.40	PC 38:7	C46H78NO8P	[M+H]+	
804.55267	804.5538	-1.40	PE 41:7	C46H78NO8P	[M+H]+	
804.55267	804.5538	-1.40	PS dO-40:8	C46H78NO8P	[M+H]+	
804.55267	804.5538	-1.40	PS O-40:6	C46H80NO9P	[M+H-H2O]+	
804.55267	804.549	4.56	PC 34:1	C42H82NO8PNa2	[M+2Na-H]+	
804.55267	804.549	4.56	PE 37:1	C42H82NO8PNa2	[M+2Na-H]+	
804.55267	804.5572	-5.63	HexCer 36:1;O4	C42H81NO10Na2	[M+2Na-H]+	
848.55793	848.5623	-5.15	IPC 36:0;O3	C42H84NO12PNa	[M+Na]+	
**848.55793**	**848.556614**	**1.55**	**PC 38:4**	**C46H84NO8PK**	**[M+K]+**	**MS/MS (see SI Figs. 12-13)**
848.55793	848.5566	1.57	PE 41:4	C46H84NO8PK	[M+K]+	
848.55793	848.5541	4.51	PE O-42:7	C47H82NO7PNa2	[M+2Na-H]+	
**832.58252**	**832.582676**	**-0.19**	**PC 38:4**	**C46H84NO8PNa**	**[M+Na]+**	**similar distribution with K adduct ion (see SI Fig. 13)**
832.58252	832.5851	-3.10	PC 40:7	C48H82NO8P	[M+H]+	
832.58252	832.5851	-3.10	PS O-42:6	C48H84NO9P	[M+H-H2O]+	
832.58252	832.5803	2.67	PC 36:1	C44H86NO8PNa2	[M+2Na-H]+	
832.58252	832.5803	2.67	PE 39:1	C44H86NO8PNa2	[M+2Na-H]+	
832.58252	832.5885	-7.18	HexCer 38:1;O4	C44H85NO10Na2	[M+2Na-H]+	
856.58383	856.5851	-1.48	PC 42:9	C50H82NO8P	[M+H]+	
**856.58383**	**856.582676**	**1.35**	**PC 40:6**	**C48H84NO8PNa**	**[M+Na]+**	**MS/MS (see SI Fig. 14)**
856.58383	856.5827	1.32	PE 43:6	C48H84NO8PNa	[M+Na]+	
856.58383	856.5757	9.49	Hex2Cer 32:1;O2	C44H83NO13Na	[M+Na]+	
856.58383	856.591	-8.37	PI 34:0	C43H83O13P	[M+NH4]+	
856.58383	856.5828	1.20	PS O-39:1	C45H88NO9PK	[M+K]+	
**872.55752**	**872.556614**	**1.04**	**PC 40:6**	**C48H84NO8PK**	**[M+K]+**	**similar distribution with M+H and Na adduct ion (see SI Fig. 14)**
872.55752	872.5566	1.05	PE 43:6	C48H84NO8PK	[M+K]+	
872.55752	872.5496	9.08	Hex2Cer 32:1;O2	C44H83NO13K	[M+K]+	

**834.60122**	**834.600732**	**0.58**	**PC 40:6**	**C48H84NO8P**	**[M+H]+**	**similar distribution with Na and K adduct ions (see SI Fig. 14)**
834.60122	834.6007	0.62	PS O-42:5	C48H86NO9P	[M+H-H2O]+	
834.60122	834.6042	-3.57	HexCer 38:0;O4	C44H87NO10Na2	[M+2Na-H]+	
834.60122	834.5959	6.37	PC 36:0	C44H88NO8PNa2	[M+2Na-H]+	
834.60122	834.5959	6.37	PE 39:0	C44H88NO8PNa2	[M+2Na-H]+	
**854.56639**	**854.567026**	**-0.74**	**PC 40:7**	**C48H82NO8PNa**	**[M+Na]+**	**similar distribution with K adduct ion (see SI Fig. 14)**
854.56639	854.5694	-3.52	PC 42:10	C50H80NO8P	[M+H]+	
854.56639	854.5753	-10.43	PI 34:1	C43H81O13P	[M+NH4]+	
854.56639	854.5672	-0.95	PS O-39:2	C45H86NO9PK	[M+K]+	
**870.5401**	**870.540964**	**-0.99**	**PC 40:7**	**C48H82NO8PK**	**[M+K]+**	**similar distribution with Na adduct ion (see SI Fig. 14)**
870.5401	870.5443	-4.82	IPC 36:0;O3	C42H84NO12PNa2	[M+2Na-H]+	
**909.55171**	**909.549853**	**2.04**	**PI 40:6**	**C49H83O13P**	**[M-H]-**	**MS/MS (see SI Fig. 15)**
909.55171	909.5418	10.90	PG 44:8	C50H83O10P	[M+Cl]-	
907.53426	***907.534203***	***0.06***	***PI 40:7***	***C49H81O13P***	***[M-H]-***	***mass accuracy***
830.51092	830.5097	1.47	PC 37:6	C45H78NO8PK	[M+K]+	
**830.51092**	**830.509663**	**1.51**	**PE 40:6**	**C45H78NO8PK**	**[M+K]+**	**similar distribution with M-H and Na adduct ions (see SI Fig. 16)**
830.51092	830.5097	1.47	PE O-40:7;O	C45H78NO8PK	[M+K]+	
830.51092	830.5071	4.60	PC O-38:9	C46H76NO7PNa2	[M+2Na-H]+	
**814.53577**	**814.535726**	**0.05**	**PE 40:6**	**C45H78NO8PNa**	**[M+Na]+**	**similar distribution with M-H and K adduct ions (see SI Fig. 16)**
814.53578	814.5381	-2.85	PE 42:9	C47H76NO8P	[M+H]+	
814.53578	814.544	-10.09	IPC 34:0;O4	C40H80NO13P	[M+H]+	
814.53578	814.5381	-2.85	PA 44:10	C47H73O8P	[M+NH4]+	
814.53578	814.544	-10.09	PI 31:0	C40H77O13P	[M+NH4]+	
814.53578	814.5359	-0.15	PS O-36:1	C42H82NO9PK	[M+K]+	
814.53578	814.5333	3.04	PC 35:3	C43H80NO8PNa2	[M+2Na-H]+	
814.53578	814.5333	3.04	PE 38:3	C43H80NO8PNa2	[M+2Na-H]+	

**790.53819**	**790.539229**	**-1.31**	**PE 40:6**	**C45H78NO8P**	**[M-H]-**	**MS/MS (see SI Fig. 16)**
790.53819	790.5392	-1.28	PE O-40:7;O	C45H78NO8P	[M-H]-	
790.53819	790.5392	-1.28	PE dO-38:8	C43H74NO6P	[M+OAc]-	
790.53819	790.5392	-1.28	PC 38:6	C46H80NO8P	[M-CH3]-	
860.61555	860.6164	-0.99	PC 42:7	C50H86NO8P	[M+H]+	
**860.61555**	**860.613976**	**1.83**	**PC 40:4**	**C48H88NO8PNa**	**[M+Na]+**	**similar distribution with M+H and K adduct ions (see SI Fig. 17)**
860.61555	860.614	1.80	PE 43:4	C48H88NO8PNa	[M+Na]+	
860.61555	860.6116	4.59	PC 38:1	C46H90NO8PNa2	[M+2Na-H]+	
860.61555	860.6116	4.59	PE 41:1	C46H90NO8PNa2	[M+2Na-H]+	
860.61555	860.6198	-4.94	HexCer 40:1;O4	C46H89NO10Na2	[M+2Na-H]+	
876.58919	876.5936	-5.03	IPC 38:0;O3	C44H88NO12PNa	[M+Na]+	
**876.58919**	**876.587914**	**1.46**	**PC 40:4**	**C48H88NO8PK**	**[M+K]+**	**similar distribution with M+H and Na adduct ions (see SI Fig. 17)**
838.63135	838.632	-0.78	PS O-42:3	C48H90NO9P	[M+H-H2O]+	
**838.63135**	**838.632032**	**-0.81**	**PC 40:4**	**C48H88NO8P**	**[M+H]+**	**similar distribution with Na and K adduct ions (see SI Fig. 17)**
**814.51476**	**814.514749**	**0.01**	**PE-P (40:6)**	**C45H78NO7PK**	**[M+K]+**	**similar distribution with M-H ion (see SI Fig. 18)**
898.57297	***898.572264***	***0.79***	***PC 42:7***	***C50H86NO8PK***	***[M+K]+***	***mass accuracy***
898.57297	898.5756	-2.93	IPC 38:0;O3	C44H88NO12PNa2	[M+2Na-H]+	
916.52413	***916.525313***	***-1.29***	***PC 44:12***	***C52H80NO8PK***	***[M+K]+***	***mass accuracy***
916.52413	916.5312	-7.71	Am-Hex-PE 34:2	C45H84NO13PK	[M+K]+	
**774.54509**	**774.544314**	**1.00**	**PE-P(40:6)**	**C45H78NO7P**	**[M-H]-**	**MS/MS (see SI Fig. 18)**
774.54509	774.5443	1.02	PC O-38:7	C46H80NO7P	[M-CH3]-	
804.59169	***804.591265***	***0.53***	***PE O-42:6***	***C47H84NO7P***	***[M-H]-***	***mass accuracy***
804.59169	804.5843	9.18	HexCer 36:1;O4	C42H81NO10	[M+Formate]-	
804.59169	804.5995	-9.71	DGTS 36:4	C46H81NO7	[M+Formate]-	
804.59169	804.5843	9.18	HexCer 35:1;O4	C41H79NO10	[M+OAc]-	

725.51223	***725.51268***	***-0.62***	***PA 38:3***	***C41H75O8P***	***[M-H]-***	***mass accuracy***
725.51223	725.5127	-0.65	PEth 36:3	C41H75O8P	[M-H]-	
725.51223	725.5209	-11.95	MGDG 32:2	C41H74O10	[M-H]-	
724.5298	***724.528664***	***1.57***	***PE O-36:4***	***C41H76NO7P***	***[M-H]-***	***mass accuracy***
724.5298	724.5369	-9.80	HexCer 35:3;O3	C41H75NO9	[M-H]-	
**812.54422**	**812.544708**	**-0.60**	**PS 38:3**	**C44H80NO10P**	**[M-H]-**	**MS/MS (see Fig. 15)**
812.54422	812.5367	9.25	PE O-40:6	C45H80NO7P	[M+Cl]-	
812.54422	812.5447	-0.59	PC 35:4	C43H78NO8P	[M+Formate]-	
812.54422	812.5447	-0.59	PE 38:4	C43H78NO8P	[M+Formate]-	
812.54422	812.5447	-0.59	PE O-38:5;O	C43H78NO8P	[M+Formate]-	
812.54422	812.5447	-0.59	PC 34:4	C42H76NO8P	[M+OAc]-	
812.54422	812.5447	-0.59	PE 37:4	C42H76NO8P	[M+OAc]-	
**768.55537**	**768.554879**	**0.64**	**PE 38:3**	**C43H80NO8P**	**[M-H]-**	**MS/MS**
768.55537	768.5551	0.35	DGTS 34:3	C44H79NO7	[M+Cl]-	
768.55537	768.5631	-10.06	HexCer 35:3;O2	C41H75NO8	[M+OAc]-	
876.58925	876.5971	-8.96	PS 39:1	C45H86NO10P	[M+Formate]-	
876.58925	876.5971	-8.96	PS 38:1	C44H84NO10P	[M+OAc]-	
**876.58925**	**876.587622**	**1.86**	**SHexCer(t40:2)**	**C46H87NO12S**	**[M-H]-**	**MS/MS**
918.63674	918.6441	-8.01	PS 42:1	C48H92NO10P	[M+Formate]-	
918.63674	918.6441	-8.01	PS 41:1	C47H90NO10P	[M+OAc]-	
**918.63674**	**918.634572**	**2.36**	**SHexCer(t43:2)**	**C49H93NO12S**	**[M-H]-**	**MS/MS**
**862.60735**	**862.608357**	**-1.17**	**SHexCer (d40:1)**	**C46H89NO11S**	**[M-H]-**	**MS/MS**
862.60735	862.6098	-2.84	PC 39:2	C47H90NO8P	[M+Cl]-	
862.60735	862.6098	-2.84	PE 42:2	C47H90NO8P	[M+Cl]-	
860.59401	860.592707	1.51	PC 39:3	C47H88NO8P	[M+Cl]-	
860.59401	860.5942	-0.22	PE 42:3	C47H88NO8P	[M+Cl]-	
860.59401	860.6022	-9.52	PS O-39:2	C45H86NO9P	[M+Formate]-	
860.59401	860.6022	-9.52	PS O-38:2	C44H84NO9P	[M+OAc]-	
**860.59401**	**860.592707**	**1.51**	**SHexCer(d40:2)**	**C46H87NO11S**	**[M-H]-**	**MS/MS**
864.63357	864.6324	1.35	IPC 40:1;O2	C46H90NO11P	[M+H]+	
864.63357	864.6407	-8.25	Hex2Cer 34:0;O2	C46H89NO13	[M+H]+	
864.63357	864.6324	1.35	IPC 40:0;O3	C46H92NO12P	[M+H-H2O]+	
864.63357	864.6325	1.24	HexCer 36:1;O	C48H91NO9K	[M+K]+	
**864.63357**	**864.632541**	**1.19**	**HexCer(t42:2)**	**C48H91NO9K**	**[M+K]+**	**similar distribution with Na adduct ion (see SI Fig. 18)**
864.63357	864.6243	10.72	PC O-40:3	C48H92NO7PK	[M+K]+	

864.63357	864.6429	-10.79	PS dO-40:0	C46H94NO8PNa2	[M+2Na-H]+	
848.65969	848.6528	8.12	PC O-42:6	C50H90NO7P	[M+H]+	
848.65969	848.6528	8.12	PC 42:4	C50H92NO8P	[M+H-H2O]+	
**848.65969**	**848.658604**	**1.28**	**HexCer(t42:2)**	**C48H91NO9Na**	**[M+Na]+**	**MS/MS (see SI Fig. 18)**
865.65578	865.6641	-9.61	PS 40:0	C46H90NO10P	[M+NH4]+	
**865.65578**	**865.655934**	**-0.18**	**SM(d43:2)**	**C48H95N2O6PK**	**[M+K]+**	**MS/MS (see SI Fig. 19)**
849.68236	849.6919	-11.23	TG 48:1	C51H96O6Na2	[M+2Na-H]+	
**849.68237**	**849.681996**	**0.44**	**SM(d43:2)**	**C48H95N2O6PNa**	**[M+Na]+**	**similar distribution with M+H and K adduct ions (see SI Fig. 19)**
827.7002	827.7	0.24	PC dO-40:4	C48H92NO6P	[M+NH4]+	
827.7002	827.7083	-9.79	HexCer 42:2;O2	C48H91NO8	[M+NH4]+	
**827.7003**	**827.700052**	**0.30**	**SM(d43:2)**	**C48H95N2O6P**	**[M+H]+**	**similar distribution with Na and K adduct ions (see SI Fig. 19)**
**825.62416**	**825.624634**	**-0.57**	**SM(d40:1)**	**C45H91N2O6PK**	**[M+K]+**	**similar distribution with M+H and Na adduct ion (see SI Fig. 20)**
809.65097	809.6606	-11.89	TG 45:0	C48H92O6Na2	[M+2Na-H]+	
**809.65098**	**809.650696**	**0.35**	**SM(d40:1)**	**C45H91N2O6PNa**	**[M+Na]+**	**similar distribution with M+H and K adduct ion (see SI Fig. 20)**
787.66813	787.677	-11.26	HexCer 39:1;O2	C45H87NO8	[M+NH4]+	
**787.66814**	**787.668752**	**-0.78**	**SM(d40:1)**	**C45H91N2O6P**	**[M+H]+**	**similar distribution with Na and K adduct ions (see SI Fig. 20)**
741.53188	741.5405	-11.62	PA 37:0	C40H79O8PNa	[M+Na]+	
741.53188	741.5307	1.59	CerPE 37:1;O2	C39H79N2O6PK	[M+K]+	
**741.53188**	**741.530733**	**1.55**	**SM(d34:1)**	**C39H79N2O6PK**	**[M+K]+**	**MS/MS (see SI Fig. 21)**
741.53188	741.5404	-11.49	DG 42:6	C45H76O5Na2	[M+2Na-H]+	
725.55722	725.548	12.71	PA 39:2	C42H79O8P	[M+H-H2O]+	
725.55722	725.5481	12.57	DG 41:4	C44H78O5K	[M+K]+	

725.55722	725.5667	-13.07	TG 39:0	C42H80O6Na2	[M+2Na-H]+	
**725.55723**	**725.556796**	**0.60**	**SM(d34:1)**	**C39H79N2O6PNa**	**[M+Na]+**	**similar distribution with M+H and K adduct ion (see SI Fig. 21)**
703.57589	703.566	14.06	DG 44:8	C47H76O5	[M+H-H2O]+	
703.57589	703.5831	-10.25	HexCer 33:1;O2	C39H75NO8	[M+NH4]+	
**703.57589**	**703.574852**	**1.48**	**SM(d34:1)**	**C39H79N2O6P**	**[M+H]+**	**similar distribution with Na and K adduct ions (see SI Fig. 21)**

References

1 Marinetti G, Erbland J, Stotz E. The structure of pig heart plasmalogens. *Journal of the American Chemical Society* 1958; **80**: 1624-1628.

2 Rapport MM, Lerner B., Alonzo N, Franzl RE. The structure of plasmogens: II. crystalline lysophosphatidal ethanolamine (acetal phospholipide). *Journal of Biological Chemistry* 1957; **225**: 859-867.

3 Debuch H. in *Biochem. J.* P27-P27 (Portland Press, 59 Portland Place, London W1N 3AJ, UK).

4 Koch J, Watschinger K, Werner ER, Keller MA. Tricky isomers – the evolution of analytical strategies to characterize plasmalogens and plasmanyl ether lipids. *Frontiers in Cell and Develo,pmental Biology* 2022; **10**: 864716.

5 Kaya I, *et al.* Spatial lipidomics reveals brain region-specific changes of sulfatides in an experimental MPTP Parkinsons disease primate model. *NPJ Parkinson's Disease* 2023; **9**: 118.
